# Functional biomarkers derived from computed tomography and magnetic resonance imaging differentiate PDAC subgroups and reveal gemcitabine-induced hypo-vascularization

**DOI:** 10.1007/s00259-022-05930-6

**Published:** 2022-09-08

**Authors:** Irina Heid, Marija Trajkovic-Arsic, Fabian Lohöfer, Georgios Kaissis, Felix N. Harder, Moritz Mayer, Geoffrey J. Topping, Friderike Jungmann, Barbara Crone, Moritz Wildgruber, Uwe Karst, Lucia Liotta, Hana Algül, Hsi-Yu Yen, Katja Steiger, Wilko Weichert, Jens T. Siveke, Marcus R. Makowski, Rickmer F. Braren

**Affiliations:** 1 School of Medicine, Institute of Diagnostic and Interventional Radiology, Technical University of Munich, Munich, Germany; 2 Division of Solid Tumor Translational Oncology, German Cancer Consortium (DKTK, partner site Essen) and German Cancer Research Center, DKFZ, Heidelberg, Germany; 3 Bridge Institute of Experimental Tumor Therapy, West German Cancer Center, University Hospital Essen, Essen, Germany; 4 Department of Computing, Imperial College London, London, SW7 2AZ UK; 5 School of Medicine, Institute for Artificial Intelligence in Medicine and Healthcare, Technical University of Munich, Munich, Germany; 6 School of Medicine, Department of Nuclear Medicine, Technical University of Munich, Munich, Germany; 7 Institute of Inorganic and Analytical Chemistry, University of Muenster, Muenster, Germany; 8 Institute of Clinical Radiology, University Hospital Muenster, Muenster, Germany; 9 Department of Radiology, University Hospital, LMU Munich, Munich, Germany; 10 School of Medicine, Clinic and Policlinic of Internal Medicine II, Technical University of Munich, Munich, Germany; 11 Comprehensive Cancer Center München, Chair for Tumor Metabolism, Klinikum rechts der Isar, Technical University of Munich, Munich, Bavaria Germany; 12 School of Medicine, Institute of Pathology, Technical University of Munich, Munich, Germany; 13 German Cancer Consortium (DKTK, partner Site Munich), Munich, Germany

**Keywords:** PDAC, CA accumulation, DCE-MRI, CT, Gemcitabine

## Abstract

**Purpose:**

Pancreatic ductal adenocarcinoma (PDAC) is a molecularly heterogeneous tumor entity with no clinically established imaging biomarkers. We hypothesize that tumor morphology and physiology, including vascularity and perfusion, show variations that can be detected by differences in contrast agent (CA) accumulation measured non-invasively. This work seeks to establish imaging biomarkers for tumor stratification and therapy response monitoring in PDAC, based on this hypothesis.

**Methods and materials:**

Regional CA accumulation in PDAC was correlated with tumor vascularization, stroma content, and tumor cellularity in murine and human subjects. Changes in CA distribution in response to gemcitabine (GEM) were monitored longitudinally with computed tomography (CT) Hounsfield Units ratio (HUr) of tumor to the aorta or with magnetic resonance imaging (MRI) ΔR_1_ area under the curve at 60 s tumor-to-muscle ratio (AUC60r). Tissue analyses were performed on co-registered samples, including endothelial cell proliferation and cisplatin tissue deposition as a surrogate of chemotherapy delivery.

**Results:**

Tumor cell poor, stroma-rich regions exhibited high CA accumulation both in human (meanHUr 0.64 vs. 0.34, *p* < 0.001) and mouse PDAC (meanAUC60r 2.0 vs. 1.1, *p* < 0.001). Compared to the baseline, in vivo CA accumulation decreased specifically in response to GEM treatment in a subset of human (HUr −18%) and mouse (AUC60r −36%) tumors. Ex vivo analyses of mPDAC showed reduced cisplatin delivery (GEM: 0.92 ± 0.5 mg/g, vs. vehicle: 3.1 ± 1.5 mg/g, *p* = 0.004) and diminished endothelial cell proliferation (GEM: 22.3% vs. vehicle: 30.9%, *p* = 0.002) upon GEM administration.

**Conclusion:**

In PDAC, CA accumulation, which is related to tumor vascularization and perfusion, inversely correlates with tumor cellularity. The standard of care GEM treatment results in decreased CA accumulation, which impedes drug delivery. Further investigation is warranted into potentially detrimental effects of GEM in combinatorial therapy regimens.

**Supplementary Information:**

The online version contains supplementary material available at 10.1007/s00259-022-05930-6.

## Introduction

Pancreatic ductal adenocarcinoma (PDAC) remains one of the deadliest tumor diseases worldwide [1], with poor prognosis (5 years survival for all stages is only 10%) [2] and low probability for curative surgery (< 20%) [3]. The two major reasons for treatment failure of PDAC are late diagnosis and a highly complex tumor microenvironment that reduces therapeutic effects. The PDAC microenvironment consists of diverse populations of embedded cancer and other associated cells (i.e., fibroblastic, stellate, endothelial, neuronal, immune), remaining ducts and substantial extracellular matrix.

PDACs are sparsely vascularized and, due to abundant matrix formation, often exhibit elevated intra-tumoral pressure and thus vascular collapse [4, 5]. Poor perfusion of PDAC has a significant clinical impact, as it contributes to reduced drug delivery and the development of drug resistance. Therefore, several agents targeting the stromal compartment have been developed and applied in clinical trials [6, 7], mostly in combination with gemcitabine (GEM), which has remained the standard of care treatment for decades [8]. Unfortunately, none of the tested combinations improved clinical outcome, including targeting of hyaluronic acid (HA) to reduce intra-tumoral pressure and inhibition of the Hedgehog pathway to block interactions between tumor and stromal cells [9–11]. Hence, despite intensive research and a great number of clinical trials, management of patients with PDAC still lacks personalized protocols and instead relies on chemotherapy [7]. The two first-line chemotherapeutic options for patients with advanced PDAC are a combination of four cytotoxic agents (i.e. fluorouracil, leucovorin, irinotecan and oxaliplatin; mFOLFIRINOX) [12] or a combination of nab-paclitaxel (Abraxane, A) and GEM [13].

Recent studies have shown that GEM therapy of PDAC affects both cancer cells and the tumor microenvironment, including vascularization. In particular, GEM has been reported to accumulate in stroma rich tumors [14]. Preclinical investigation in clinically relevant endogenous mouse models of PDAC revealed that active metabolites of GEM are captured by cancer-associated fibroblasts (CAFs), pancreatic stellate cells (PSC), and M2-polarized macrophages, which possibly reduces its cytotoxic effect on the cancer cells [15, 16]. Inconclusive results have been reported for GEM regarding endothelial cells and tumor vascularization. For example, human endothelial cells were highly sensitive to low concentrations of GEM in vitro [17]. Reduced perfusion was noted after GEM treatment in a human PDAC xenografts as well as in endogenous murine tumors harboring *KRAS* and *TP53* mutations [18]. However, other reports have shown increased perfusion in response to GEM treatment in murine and human tumors in vivo [19–21].

Increasing appreciation of the molecular and histopathological heterogeneity in PDAC has led to careful re-evaluation of imaging-derived biomarkers for non-invasive differentiation of PDAC subgroups and detection of individual therapy response. Imaging is routinely performed after systemic injection of contrast agents (CA) to visualize its relative regional accumulation as a surrogate of local blood supply and tissue composition. This clinical approach, to some extent, disregards the complexity of the underlying systemic (i.e. CA injection rate, heart rate, blood pressure, kidney function, etc.) and regional (i.e. vascularity, perfusion, permeability, tissue composition, etc.) determinants of CA biodistribution, which are greatly simplified therein. Computed tomography (CT) is the standard method for diagnosis and response monitoring of PDAC patients in clinical routine. CT is performed statically, after intravenous injection of iodine-based CA, which accumulates locally and causes differences in X-ray attenuation, quantified in Hounsfield Units (HU). Magnetic resonance imaging (MRI) is typically performed pre-clinically. In dynamic contrast enhanced (DCE)-MRI, a series of T_1_-weighted images, or T_1_ maps, is acquired before, during, and after intravenous injection of Gd^3+^-based CAs with time resolution of 3–5 s. Although CT and DCE-MRI are different imaging techniques, both have been used to describe regional tissue morphology and physiology, including vascularity and perfusion and changes thereof [22–24]. In PDAC, HU ratios (HUr) of tumor tissue, normalized to the aorta or tumor adjacent pancreas, have shown a positive correlation with desmoplastic stroma and a negative correlation with tumor cellularity and patient survival [14, 25–27]. For DCE-MRI, commonly used parameters are the volume transfer constant (K^trans^) and the extravascular extracellular volume fraction (v_e_) determined using Tofts pharmacokinetic modeling [28]. Differences in tumor perfusion and permeability, measured by K^trans^, were also correlated with biological variations in tissue composition, response to therapy and clinical outcome in PDAC [29–32]. However, such modeling introduces a high level of complexity and uncertainty. In contrast, area under the time–to–signal curve, e.g., 60 or 90 s after the arrival of the contrast bolus (iAUC60, iAUC90), has been used as a less accurate albeit feasible alternative. AUCs derived from signal intensities in T_1_-weighted images or CA concentrations calculated from T_1_ (=1/R_1_) maps are parameters that, similarly to CT HU, reflect a combination of blood flow, vessel size and density, permeability, and functionality of the micro-vascular network [22, 33]. For example, AUC60 has been correlated directly to perfusion measurements and K^trans^ estimations in murine and human studies [23, 34] and suggested as a surrogate perfusion biomarker. In a study on differently vascularized tumors, iAUC90 was reported to be even more sensitive to vascular changes under anti-angiogenic treatment than K^trans^ [22]. In addition, AUC is widely established for perfusion measurement in preclinical trials of small animals, where measuring the arterial input function (AIF) is particularly difficult and error prone [35]. To account for inter-subject variability, both imaging techniques, with their respective parameters, rely on normalization (i.e., the aorta in patient CT and spinal muscle in mouse MRI) [27, 36].

In this study, our aim was to establish imaging biomarkers based on CA accumulation and investigate their potential to detect differences in tissue composition and changes caused by chemotherapy in PDAC. For patient studies, we analyzed CA accumulation in routine pre-operative CT (HUr) and DCE-MRI (signal intensity of tumor to aorta ratios (SI_t/a_)). To investigate the effects of chemotherapy on CA accumulation, we used clinically relevant murine models and the standard preclinical imaging technique DCE-MRI (AUC60r). Using murine histopathological samples, we investigated GEM treatment effects on the cellular level. Finally, we analyzed GEM treatment effects on CA accumulation in human PDAC.

## Materials and methods

### Clinical CT and MR imaging protocol

CT imaging patient data were included when following criteria were met: contrast enhanced in portal venous (PV) phase 70 s after injection of CA (Ultravist®-370 Bayer, 70 mL, followed by a 30 mL saline chaser) in inspiratory breath hold, reformatted slice thickness at most 3 mm, availability of axial, sagittal, and coronal reformations in medium-hard kernels. Mean CA enhancement in the PV phase was calculated from 5 mm ROI in tumor and the aorta to build HUr as described in supplementary methods.

Clinical DCE-MRI data were included when acquired as T_1_-weighted images with spectral fat saturation in the early venous phase of 50–70 s and late venous phase of 100–180 s after administration of contrast agent (Gd-DOTA, Magnevist® at a concentration of 0.1 mmol/kg). Only data with corresponding CT and histological analysis were included in the study. ROIs were placed into the CT-corresponding MRI regions within the tumors as shown in Figure S1A, the aorta, and muscle to build signal intensity tumor/aorta (SI_t/a_) or tumor/muscle (SI_t/m_) ratios.

### Description of patient cohorts and data analysis

**Fig. 1:**
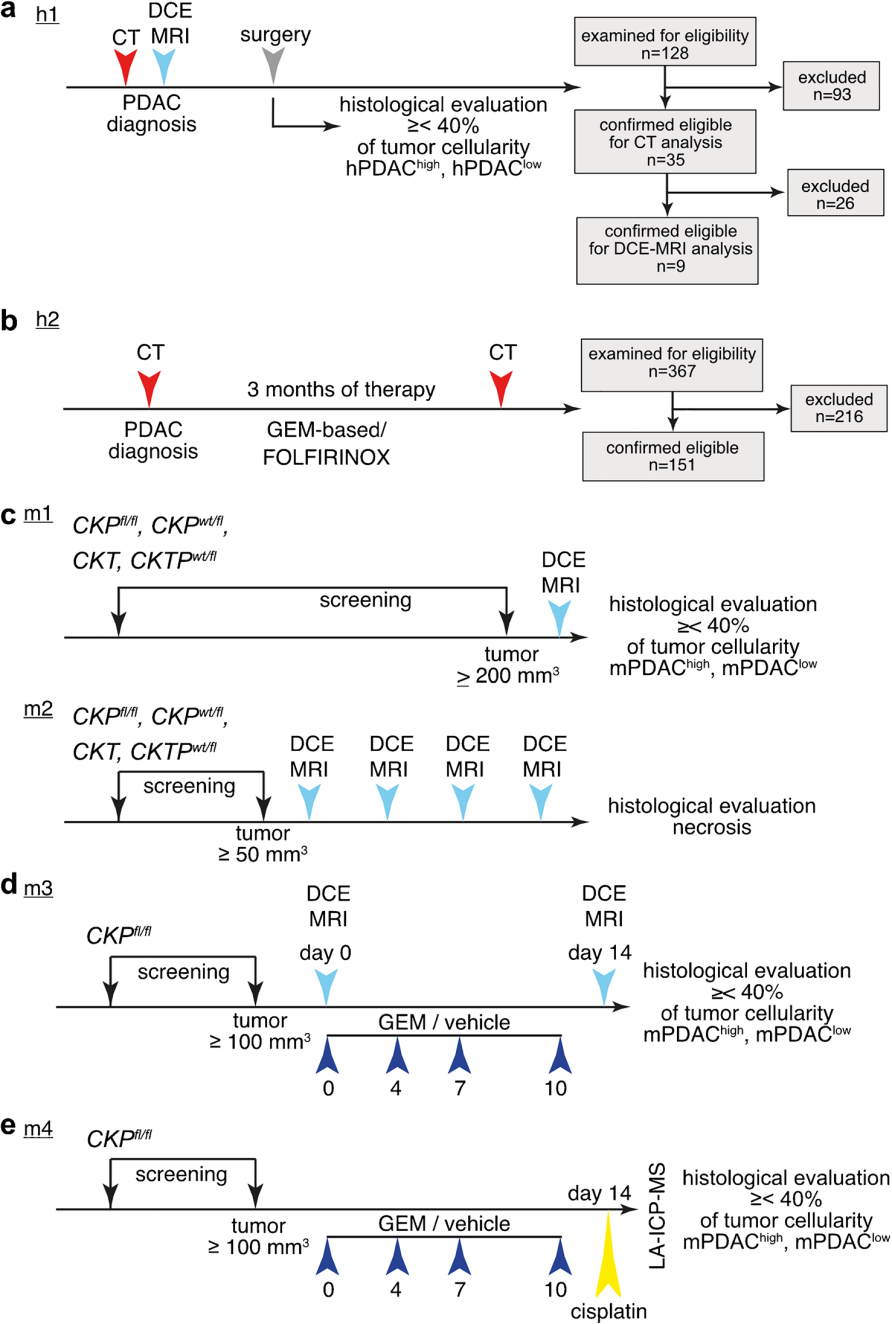
Time lines of human and murine imaging. **a** Study h1: therapy-naïve PDAC (*n* = 35) patients received CT prior surgical resection. Nine patients also received a preoperative DCE-MRI that was used in this study. Ninety-three patients were excluded due to following reasons: absence of PDAC in final histopathologic report (*n* = 34), insufficient imaging protocol (*n* = 35), prior treatment (*n* = 18), death within 6 weeks after operation (*n* = 6). **b** Study h2: PDAC patients (*n* = 151) received CT before and approximately 3 months after initiation of a GEM-based (*n* = 94) or FOLFIRINOX (*n* = 57) chemotherapy. Two hundred sixteen patients were excluded due to following reasons: absence of PDAC in final histopathologic report (*n* = 57), insufficient imaging protocol (*n* = 121), prior treatment (*n* = 38). **c** Study m1: Tumors (n_ROI _= 56) grown in *CKP*^*fl/fl*^, *CKP*^*wt/fl*^, *CKT*, or *CKTP*^*wt/fl*^ animals were weekly monitored by T2w-MRI and DCE-MRI (end point: *n* = 56, study m1; thereof longitudinally: *n* = 10, study m2). **d** Study m3: tumors in *CKP*^*fl/fl*^ animals were treated with GEM (*n* = 15) or vehicle (*n* = 14) and subjected to T2w- and DCE-MRI before and after treatment. **e** Study m4: tumors in *CKP*^*fl/fl*^ animals were treated with GEM (*n* = 7) or vehicle (*n* = 7) and subjected to a single dose of cisplatin before sacrifice. After sacrifice, tumors were grouped according to cellularity based on H&E staining

This study was designed as a retrospective observational cohort study. All patients were referred to the radiology department for suspected pancreatic cancer. Imaging data of patients (human = h) were included as indicated in Fig. 1a.

***Study h1:*** To analyze the relationship of histopathological tissue composition and CA accumulation in PDAC, 128 patients, who underwent pancreaticoduodenectomy between September 2016 and March 2019, were considered eligible (Fig. 1a). Patients without histological confirmation of PDAC, incomplete imaging protocol or technically insufficient imaging (due to e.g. motion artifacts or stent placement), neoadjuvant chemotherapy, resection prior to enrolment, or who were dead within the first 6 weeks of follow-up (to limit bias from postoperative complications) were excluded. In total, 35 untreated patients (18 male aged 69±9.3 years; 17 female aged 71±7.8 years) were enrolled for the CT study and a subset of 9 patients for the MRI study. Preoperative CT data were carefully correlated by two experienced pathologists and two experienced radiologists with all available H&E stained histological samples based on the pathology reports. Up to two distinct CT ROIs per patient were correlated with corresponding H&E slices using morphological landmarks (i.e., main pancreatic or bile duct, location of main arteries and venues, cysts, duodenum). The CT analysis was transferred to the MRI data as described in Supplementary Methods. Patients were grouped as hPDAC^low^ when the amount of tumor cells determined visually by pathologist was lower than 40% in a microscope field of view of 1.5 mm diameter (20× objective, Olympus BX35 light microscope). All other ROIs, containing ≥ 40% of tumor cells, were defined as hPDAC^high^. ***Study h2***: To analyze the effect of GEM- or FOLFIRINOX-based therapy on tumor CA accumulation in CT, as a surrogate for tumor vascularization and perfusion, 367 patients were considered eligible (Fig. 1b). Patients with an incomplete imaging protocol, prior therapy, or missing final diagnosis of PDAC were excluded. In total, 151 patients with histopathologically proven diagnosis of locally advanced PDAC between January 2010 and December 2017, who received GEM monotherapy or GEM in combination with nab-Paclitaxel (summarized as GEM-based therapy, *n *= 94) or FOLFIRINOX (*n* = 57) and a standardized in-house CT exam before (3±1.1 weeks) and after (12±2.5 weeks) therapy onset, were retrospectively evaluated. This evaluation was done as described for study h1. Analyses were adjusted to take the larger volume into account, i.e., 5 ROIs in tumor or aorta were analyzed and summarized. For the assessment of CA accumulation change under chemotherapy, the following calculation was performed: Change in CA Accumulation % = (Ratio_post_/Ratio_pre_) * 100% – 100%. Clinical data are summarized in Table 1. Data for survival analysis was collected until October 31st, 2020.

**Table 1:** Distribution of clinical parameters in GEM-based and FOLFIRINOX-treated patients. *CTx*, chemotherapy, *T*, T-status, *N*, lymph node infiltration, *M*, metastases, *G*, grading, *CA*, carbohydrate antigen. Data are given as respective stage (e.g., cT 1): number of patients (e.g., 0). Chi-square statistics was performed for comparison.

Parameter	FOLFIRINOX (n=57)	Gemcitabine-based (*n* = 94)	*p*
Age (mean [years] (std. dev.))	62 (± 9)	70 (±10)	<0.0001
Sex	Female: 23, male: 34	Female: 46, male: 48	0.3178
Neoadjuvant/palliative CTx	19/38	16/78	
cT	1: 0, 2: 6, 3: 8, 4: 24	1: 1, 2: 16, 3: 15, 4: 46	0.882
pT	1: 1, 2: 1; 3: 13, 4: 4	1: 2, 2: 5, 3: 7, 4: 2	0.154
cN	0: 26, 1: 7, 2: 5	0: 41, 1: 19, 2: 18	0.2488
pN	0: 9, 1: 8, 2: 2	0: 4, 1: 11, 2: 1	0.2878
cM	0: 30, 1: 27	0: 38, 1: 56	0.4905
pM	0: -, 1: 13	0: -, 1: 32	
G	G1: 2, G2: 12, G3: 13 Grading not available: *n* = 30	G1: 3, G2: 22, G3: 20 Grading not available: *n* = 49	0.935
CA 19-9 U/l (mean, std. dev.)	975 (±1777) CA 19-9 available: *n* = 36	3447 (±7303) CA 19-9 available: *n* = 76	0.0619

### Genetically engineered mouse models (GEMM)

Analyzed lesions (*n* = 56) were derived from several experimental models (GEMM) of following genotypes: *Ptf1a*^*wt/cre*^*(C)Kras*^*wt/G12D*^*(K)Ela-Tgfa(T) *n = 6, *CK;p53(P)*^*wt/fl*^ n* =* 14, *CKP*^*fl/fl*^ n = 19, *CKTP*^*wt/R172H*^/*CKTP*^*wt/fl*^ n = 7). These genetic strains have been described elsewhere [37] and are widely used in preclinical studies. Only mPDAC lesions with a grading of 1– 4 were considered and acinus cell carcinomas were excluded.

### Description of murine studies

Murine imaging data was collected prospectively. Time lines of murine (m) imaging are shown in Fig. 1c, d. ***Study m1:*** to analyze tumor CA accumulation in untreated mice, regular screening was performed by T2-weighted (T2w)-MRI at the time of the predicted tumor onset, depending on the genotype of the animal. Fifty-six tumors (≥ 200 mm^3^) in 46 animals were examined by DCE-MRI prior to sacrifice (Fig. 1c upper panel). Of these, 10 tumors were monitored longitudinally by DCE-MRI (***study m2***, Fig. 1c lower panel). After sacrifice, mPDAC were grouped in accordance with hPDAC analyses, as mPDAC^low^ when the amount of tumor cells in the H&E stained ROI (as described below) corresponding to imaging was lower than 40%, and as mPDAC^high^ for all other ROIs, containing ≥ 40% of tumor cells. ***Study m3***: to analyze changes in tumor CA accumulation in response to GEM treatment, tumor bearing *CKP*^*fl/fl*^ animals were treated with GEM (13 animals, 15 tumors, 120 mg/kg) or vehicle (10 animals, 14 tumors, 0.9% NaCl) on day 0, 3, 7, and 10, as previously described [38] and monitored by T2w- and DCE-MRI before (day 0) and after (day 14) treatment (Fig. 1d). ***Study m4***: to analyze the effect of GEM on cisplatin accumulation in the tumor as a surrogate of tumor vascular function, tumor bearing *CKP*^*fl/fl*^ animals were treated with GEM (3 animals, 7 tumors) or vehicle (3 animals, 7 tumors) as described above and subjected to a single i.p. injection of 7.5 mg/kg cisplatin 5 min prior to sacrifice (Fig. 1e). After death, tissues were manually perfused with 20 ml NaCl and 50 ml cold 4% PFA/PBS solution, in order to remove cisplatin from the vessels.

### DCE-MR-Imaging protocol and imaging data analysis in mice

DCE-MRI were performed on a clinical 1.5 T MRI System (Achieva 1.5 T, Philips Medical Systems, Best, The Netherlands) using a 47 mm microscopy surface coil during free breathing with fast single-shot Look-Locker based radial T_1_ mapping technique using the golden cut principle (LLGC) as previously described [39]. A bolus dose of 0.04 mmol/kg of Gd-DTPA (Magnevist®) was administered after 60 s. Fit T_1_ time curves were converted to ΔR_1_ = 1/T_1_ – 1/T_10_, where T_10_ was determined from the time-averaged ROI T_1_ in 6 frames prior to CA injection. The tumor-to-muscle ratios were calculated and presented as AUC60r. More details are given in Supplementary Methods.

### Tissue preparation, histological staining, and analysis

At least 10 axial histological slices were collected and stained with Mayer’s hemalun and eosin (H&E). The best corresponding slide was selected and manually co-registered and analyzed with in vivo imaging [40]. Available consecutive slides were stained with vascular marker CD31 (m) and Ki67 or CD34 (h) as described in Supplementary Methods.

### Statistical analysis

All statistical tests were performed using GraphPad prism version 7. All groups were tested for normal distribution using D’Agostino & Pearson omnibus and Shapiro-Wilk normality tests. Data were marked as normal (*), or skewed (#) distributed and subsequently correlated using Pearson’s or Spearman’s tests. For group comparisons, mean values and standard deviations are presented. All groups were found to be normally distributed (or assumed to be normally distributed for group size <10) and were compared using paired or unpaired Student’s *T* tests. Survival analysis was done with the log-rank test.

### Laser ablation – inductively coupled plasma – mass spectrometry (LA-ICP-MS)

LA-ICP-MS imaging is described within Supplementary Methods.

## Results

### CA accumulation in a tumor negatively correlates with tumor cellularity in human and murine PDAC

**Fig. 2:**
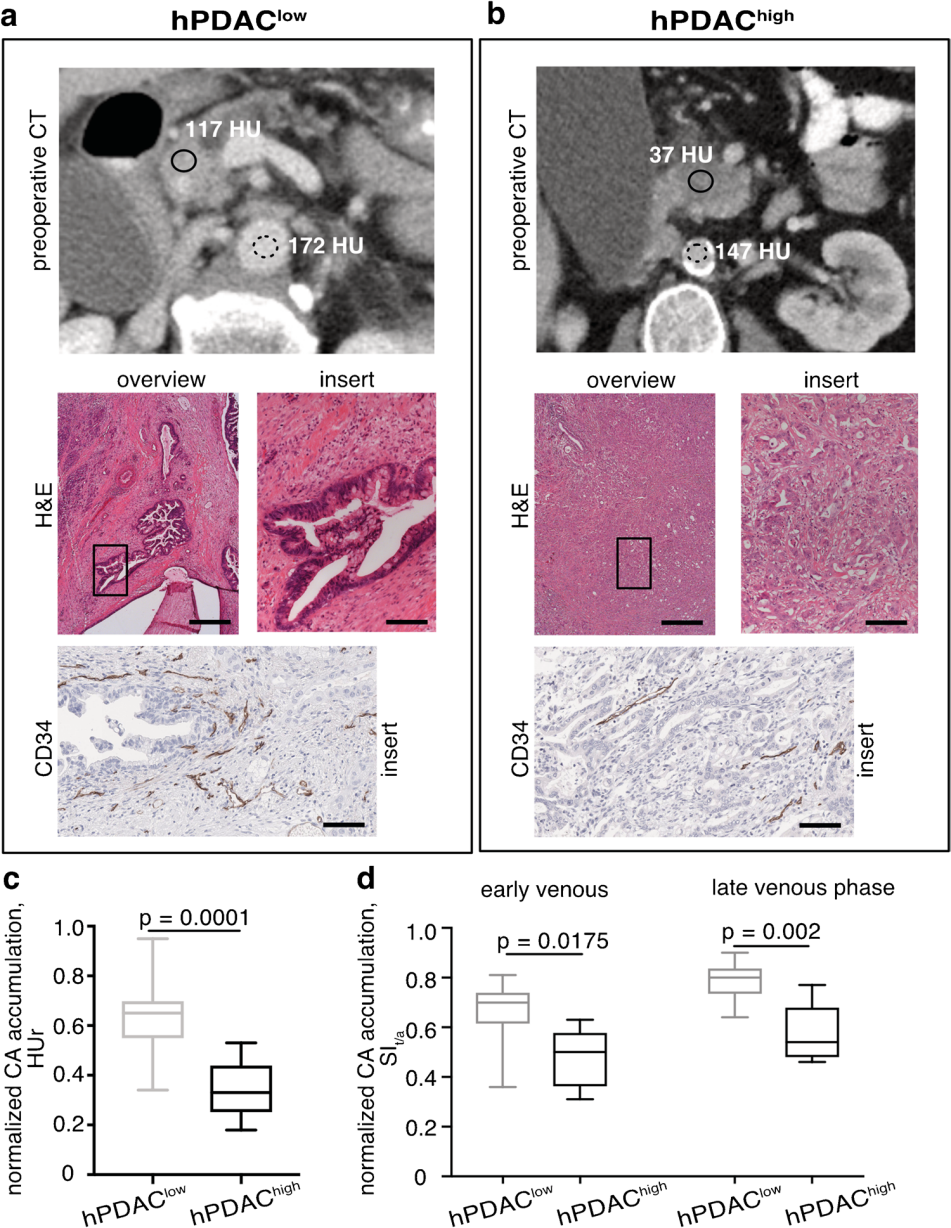
Distinct CA accumulation pattern in hPDAC. **a, b** Inter-tumoral differences in CA accumulation and tissue composition of hPDAC. Two representative examples of PDAC patients with high (**a**, hPDAC^low^, HUr = 0.68) and low (**b**, hPDAC^high^, HUr = 0.25) CA accumulation. From top to bottom: preoperative CT enhancement pattern (PV phase) with ROI of tumor (solid line) and the aorta (dotted line), window settings (level/width): 50/350 for conventional CT; examples of photomicrographs of H&E overview (scale bar 5 mm); and magnified (scale bar 100 μm) histology sections stained for H&E and CD34 staining. **c** Box-and-whisker plot of CA accumulation values for histologically confirmed hPDAC^low^ and hPDAC^high^ regions derived from PV phase of CT imaging. **d** Box-and-whisker plot of CA accumulation values for histologically confirmed hPDAC^low^ and hPDAC^high^ regions derived from early (70 s) and late (100–180 s) venous phase of DCE-MRI calculated as SI_t/a_ ratios

To test whether regional CA accumulation reflects differences in tissue composition in the patient cohort, we retrospectively correlated clinical routine H&E-stained tumor slides and HUr in pre-operative CT scans of 35 therapy-naïve, primarily resected PDAC patients (study h1, Fig. 1a). We modified our previous stratification [27, 37] and defined two morphological groups of different tumor cellularity, described as hPDAC^low^ with < 40% of cancer cells and hPDAC^high^ with ≥ 40% of cancer cells within the ROI. Examples of two tumors are shown in Fig. 2a, b: one exhibiting high CA accumulation and few tumor cells (hPDAC^low^) and one of the reverse case of low CA accumulation and many tumor cells (hPDAC^high^). Regional CA accumulation values revealed significant differences for the two cellularity groups (hPDAC^low^: meanHUr = 0.64 ± 0.13, *n* = 26, and hPDAC^high^: meanHUr = 0.34 ± 0.11 *n* = 23, Fig. 2c, *p* = .0001), confirming an inverse relationship between CA accumulation and tumor cellularity. In addition, regions of high tumor cellularity also revealed lower vascularity and vice versa, as shown by staining of CD34 in Fig. 2a, b, suggesting that increased tumor cellularity correlates with reduced vascularity and that CA accumulation measured by HUr can serve as surrogate biomarker for tumor perfusion.

Since clinically used CT and pre-clinically used DCE-MRI are performed with different contrast agents that may distribute differently in PDAC, we identified a subset of 9 patients from the above described cohort that were imaged with both modalities and additionally calculated SI_t/a_ and SI _t/m_ ratios of 15 ROIs for the early and late venous phase of Gd-based CA enhancement detected by MRI. Similar to CT, regional SI_t/a_ differed significantly between the cellularity groups (i.e., early venous phase, hPDAC^low^: mean SI_t/a _= 0.66 ± 0.13, *n* = 9, and hPDAC^high^: mean SI_t/a _= 0.48 ± 0.12, *n* = 6, *p* = 0.018, Fig. 2d). The same observation was true for SI_t/m_ in both venous phases, as shown in Figure S1B. Furthermore, correlation was as good in the early venous phase between the HUr and SI_t/a_ within the same patients (*r* = 0.66*, *n* = 15, *p* = 0.008, Figure S1C), supporting the assumption that both imaging modalities capture differences in tumor cellularity.

**Fig. 3:**
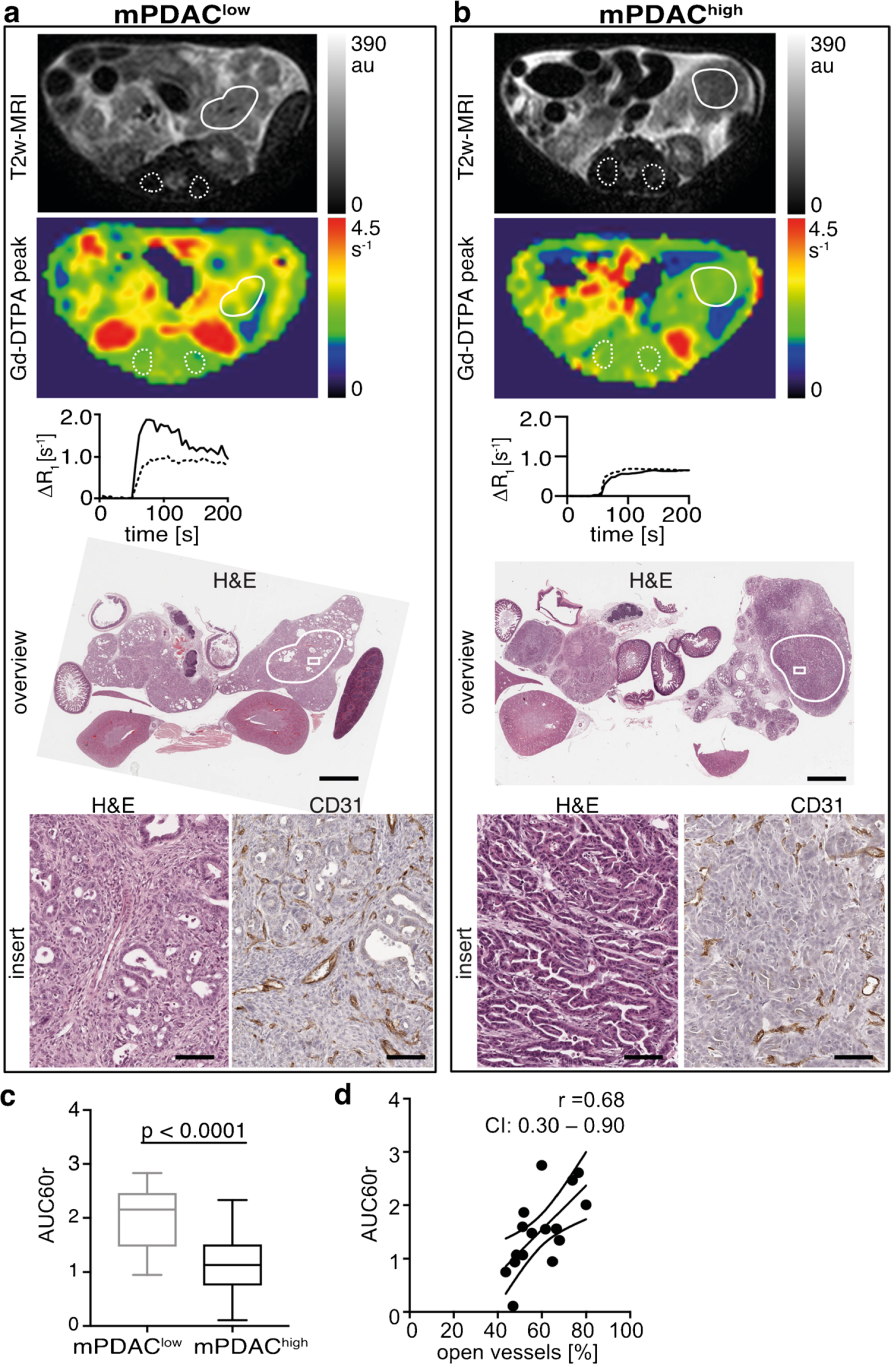
CA accumulation in mPDAC depends on tissue composition similarly to hPDAC, representing a good preclinical model. Two representative samples of well (**a**) and poorly (**b**) perfused mPDAC. From top to bottom: T2w MRI with tumor (solid line) and muscle (dotted line) ROIs; corresponding R_1_-map at peak; ΔR_1_ time curve of tumor (solid line) and muscle (dotted line); photomicrographs of corresponding H&E overview (scale bar 1 mm); and magnified (scale bar 50 μm) histology sections stained for H&E and vascular marker CD31. **c** mPDAC revealed similar to hPDAC dependency of tumor perfusion on tissue composition shown as AUC60r. Box-and-whisker plot of normally distributed data. **d** Tumor perfusion-related parameter AUC60r reveals strong correlation with percentage of open vessels in mPDAC. Regression line, Spearman correlation coefficient (*r*), and confidence interval (CI) are shown

Since late stages of PDAC are rarely operable, most imaging studies in human PDAC are performed without or with insufficient (low sample size) histopathological correlation. In order to overcome this impendent, we performed prospective imaging studies with thoroughly correlated histopathological samples in a variety of GEMM that represent a broad range of histo-morphologies and disease stages of PDAC: *Ptf1a*^*wt/cre*^*(C)Kras*^*wt/G12D*^*(K)Ela-Tgfa(T) *n = 6, *CK;p53(P)*^*wt/fl*^ n* =* 14, *CKP*^*fl/fl*^ n = 19, *CKTP*^*wt/R172H*^/*CKTP*^*wt/fl*^ n = 7 (study m1, Fig. 1c). To investigate the relationship between tumor cellularity and CA accumulation in mPDAC, we evaluated AUC60r as surrogate biomarker of tumor cellularity and in addition as surrogate biomarker of tumor vascularity. Fifty-six lesions in these treatment-naive animals were analyzed and revealed major variation in CA accumulation, corresponding histopathology and vascular staining (Fig. 3a, b). Tumors classified as mPDAC^low^ showed significantly higher CA accumulation (meanAUC60r = 2.0 ± 0.6, *n* = 29, Fig. 3c) in comparison to mPDAC^high^ (meanAUC60r = 1.1 ± 0.5, *n* = 27, *p* < 0.0001). Histopathological analyses revealed a moderate positive correlation of CA accumulation measured by AUC60r with regional tumor stroma content (*r* = 0.54#, CI: 0.32 to 0.71, *p* < 0.0001, *n* = 56, Figure S2A) and a negative correlation with the amount of tumor cells (*r* = −0.53#, CI: −0.70 to −0.30, *p* < 0.0001, *n* = 56, Figure S2B). Furthermore, mPDAC^low^ showed a higher percentage of open and therefore presumably perfused vessels per analyzed ROI (65 ± 11%) than mPDAC^high^ (50 ± 11%) as shown in Figure S2C. Overall, differences in AUC60r correlated more strongly with the percentage of open vessels (Fig. 3d, *r* = 0.68#, CI: 0.30 to 0.90, *p* = 0.005, *n* = 16) than with the absolute number of vessels per tumor region (*r* = 0.44#, CI: -0.08 to 0.77, *p* = 0.1, *n* = 16, Figure S2D), further suggesting strong relation of AUC60r to functional vessels and therefore perfusion.

Longitudinal DCE-MRI of untreated tumors (study m2, Fig. 1c) revealed short- and long-term reproducibility in stable tumors (Figure S3) and long-term decrease of AUC60r in tumors with increasing cellularity (Figure S4, tumor right). Sudden major decrease in CA accumulation occurred in verified areas of spontaneously developed tumor necrosis (Figure S3, tumor left).

### Gemcitabine reduces CA accumulation and chemotherapy delivery in mPDAC through inhibition of endothelial cell proliferation

**Fig. 4:**
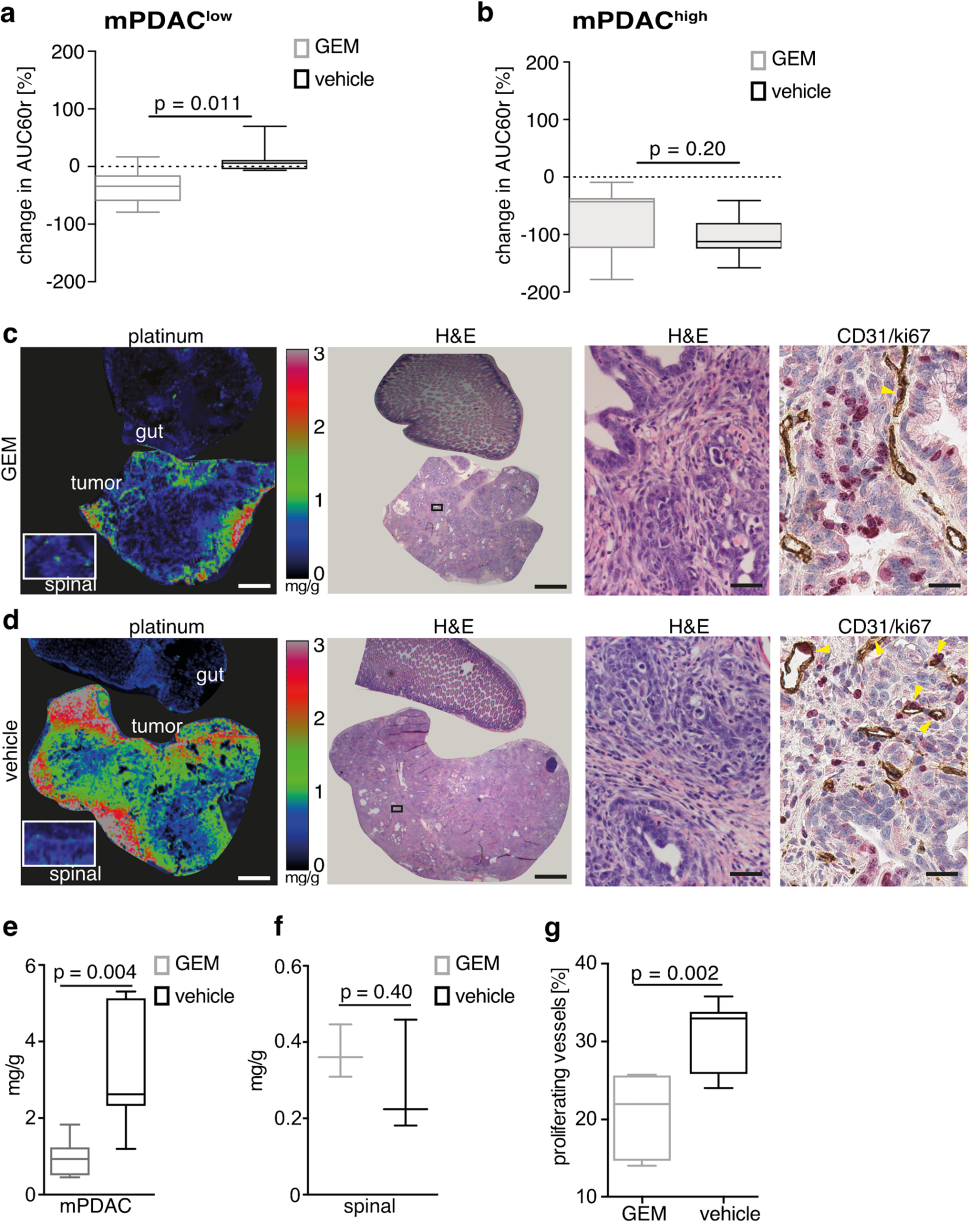
Gemcitabine treatment reduces tumor CA accumulation, drug delivery and vascular proliferation in mPDAC. **a** In mPDAC^low^ perfusion-sensitive parameter meanAUC60r was more profoundly reduced by GEM compared to vehicle treatment as shown on as Box-and-whisker plot. **b** Poorly perfused mPDAC^high^ reduce CA accumulation independent of the treatment course. **c** GEM reduces drug delivery mimicked by cisplatin injection in mPDAC. From left to right: color-coded maps showing platinum distribution in pancreatic tissue after 2 weeks of or **c** GEM or **d** vehicle treatment measured in the extracted tissue by LA-ICP-MS; corresponding photomicrographs of H&E stained overview (scale bar 1 mm), regional magnification of H&E (scale bar 100 μm), and double CD31/ki67 staining (yellow arrows show proliferating vessels, scale bar 50 μm). **e** Box-and-whisker plot of quantification of platinum concentrations in mPDAC tissue samples and **f** reference spinal tissue samples. **g** Box-and-whisker plot of quantification of endothelial cell proliferation presented as percentage of double positive (CD31 and ki67) vessels over all vessels in cross-sections

Next, we tested the effect of GEM treatment on tumor perfusion and permeability in mPDAC using AUC60r. A significant decrease in AUC60r values (meanAUC60r_pre _= 2.5 ± 0.7, meanAUC60r_post _= 1.7 ± 0.6, *p* = .0003) after GEM treatment was observed, but not after vehicle treatment (meanAUC60r_pre _= 2.2 ± 0.7, meanAUC60r_post _= 1.9 ± 1.1, *p* = 0.31). The reduction of tumor CA accumulation after GEM was very prominent in well-perfused mPDAC^low^ tumors (Fig. 4a, mean change compared to baseline: GEM −34% ± 31% vs. vehicle +13% ± 26%, *p* = 0.01) compared to poorly perfused mPDAC^high^ tumors (mean change: GEM −69% ± 60% vs. vehicle −106% ± 68%, *p* = 0.2, Fig. 4b). Intrigued by this finding, we tested small molecule delivery as a surrogate of chemotherapy delivery to murine tissue after 2 weeks of GEM therapy using laser ablation – inductively coupled plasma – mass spectrometry (LA-ICP-MS) imaging (Fig. 4c, d left panels). We found a significant decrease in cisplatin deposition, specifically in tumor tissue following the pre-treatment with GEM (Fig. 4e, GEM: 0.92 ± 0.5 mg/g, vehicle: 3.1 ± 1.5 mg/g, *p* = 0.004) compared to spinal muscle tissue (Fig. 4f, GEM: 0.37 ± 0.07 mg/g, vehicle: 0.29 ± 0.15 mg/g, *p* = 0.4). Furthermore, CD31/Ki67 double staining of corresponding tumor sections (Fig. 4c, d right panels) revealed a significant reduction in the percentage of proliferating endothelial cells in vessels that were subjected to systemic GEM treatment (GEM: 22.3 ± 4.2%, vehicle: 30.9 ± 3.9%, *p* = 0.002, Fig. 4g).

### Gemcitabine treatment reduces tumor CA accumulation in hPDAC

**Fig. 5:**
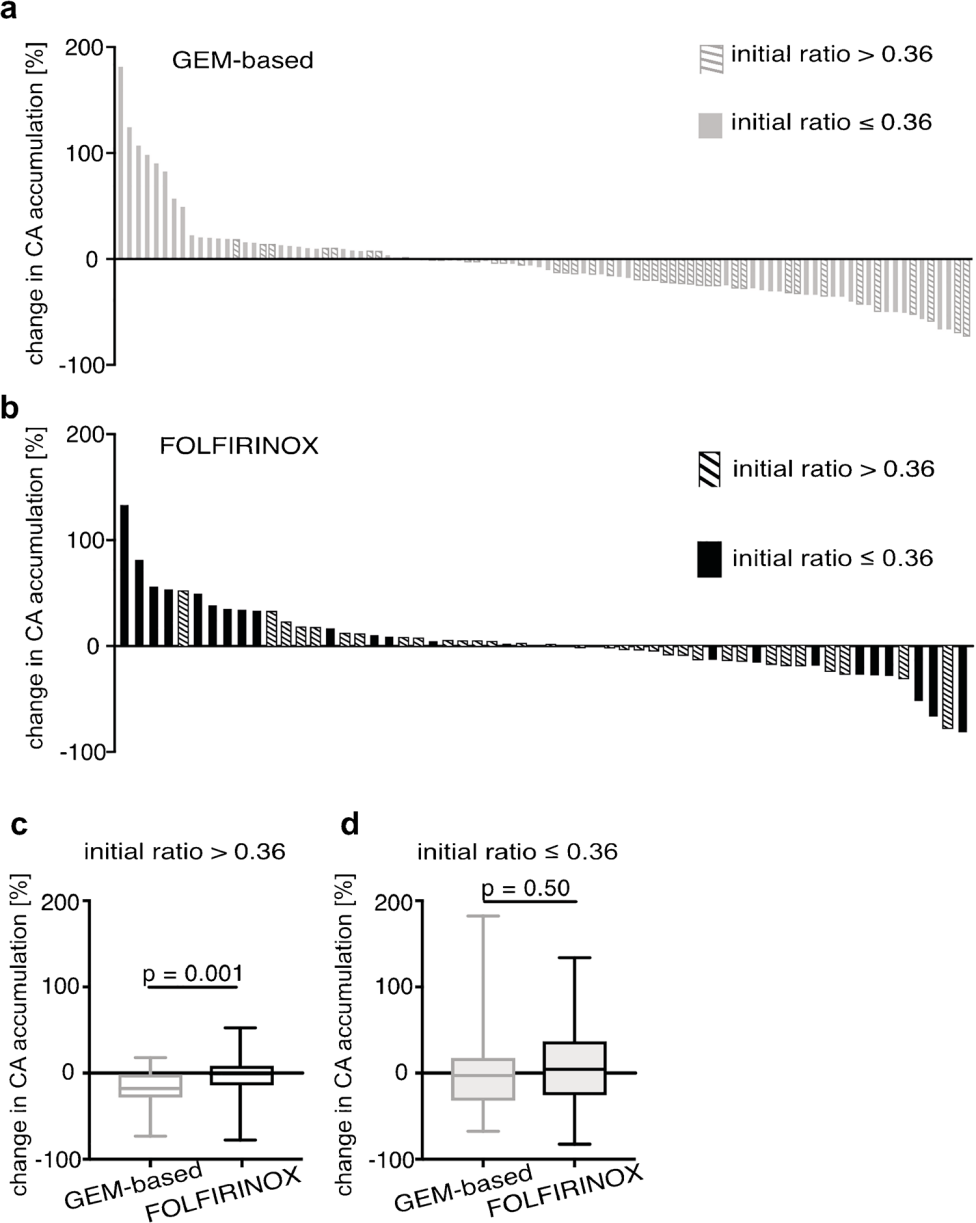
GEM-based therapy reduces CA accumulation in tumors with high initial HUr detected via CT imaging. Waterfall plots of relative change in per cent of tumor CA accumulation values in **a** GEM-based and **b** FOLFIRINOX-treated hPDAC separated by initial CI accumulation ratio (dashed bars HUr > 0.36, full bars HUr ≤ 0.36). **c** GEM-treated tumors that initially showed high CA accumulation reveal significant drop in CA accumulation compared to FOLFIRINOX treatment. Box-and-whisker plot. **d** hPDAC that initially showed low PV enhancement ratio reveal no significant differences between the two treatment regiments as shown in box-and-whisker plot

To investigate GEM effects on CA accumulation in hPDAC, we retrospectively analyzed change of CA accumulation at baseline and follow-up CT scans obtained within 3 months in 151 patients (study h2, Fig. 1b, cohort description is summarized in Table 1). Figure 5a, b shows changes observed in the individual patients represented as waterfall plots, starting at the observed most positive and finishing at the most negative change. Particularly, we were interested in the differences in change of CA accumulation between different treatment regimens for tumors with initially high HUr because these revealed most prominent changes in the murine cohort. Since imaging data of this chemotherapy-treated patient cohort could not be correlated to histopathology, the cutoff was chosen as the mean CA accumulation value of the whole population prior to therapy: meanHUr = 0.36, CI: 0.33 to 0.38. Consequently, 41 GEM and 31 FOLFIRINOX-treated patients were classified as initially well perfused (meanHUr > 0.36), and 53 GEM-based and 26 FOLFIRINOX-treated patients were classified as initially poorly perfused (meanHUr ≤ 0.36). Mirroring findings in mPDAC, a significantly larger decrease in CA accumulation was evident in tumors with initial HUr > 0.36 after GEM treatment (Fig. 5c, GEM-based: meanHUr_pre _= 0.49 ± 0.10, meanHUr_post _= 0.40 ± 0.14; mean change −18.5%, CI: −25% to −12%) compared to changes in CA accumulation in FOLFIRINOX-treated patients (meanHUr_pre _= 0.49 ± 0.09, meanHUr_post _= 0.47 ± 0.11; mean change −2.1% CI: −10% to +6%, *p* = .001). Again, as observed in mPDAC, in tumors that showed an initial low CA accumulation, no significant differences between the two treatment regimens were detected (Fig. 5d; GEM-based: meanHUr_pre _= 0.22 ± 0.08, meanHUr_post _= 0.23 ± 0.13; mean change +5%, CI: −8.5% to +18%; FOLFIRINOX: meanHUr_pre _= 0.28 ± 0.8, meanHUr_post _= 0.29 ± 0.13; mean change +8%, CI: −11% to +27%; *p* = 0.50). Observed changes in hPDAC showed no correlation with therapy response measured according to RECIST criteria, such as change in tumor diameter (GEM: *r* = 0.1#, *p* = 0.4; FOLFIRINOX: *r* = 0.1#, *p* = 0.7). As expected, patients treated with FOLFIRINOX survived longer than those treated with GEM (17 vs. 10.5 months, Figure S5A). Separation of patient based on initial HUr (≤ or > 0.36) revealed no differences in survival of FOLFIRINOX patients (18 vs 15 months, Figure S4B), however a significant difference in overall survival of GEM-treated patients (8.8 vs. 15 months, Figure S5B), confirming our hypothesis that HUr can serve as imaging biomarker for tumor aggressiveness.

## Discussion

Tumor morphology, physiology, and clinical outcome reveal substantial heterogeneity in PDAC with high tumor cellularity as an indicator of poor prognosis [14, 25, 37]. Consequently, to facilitate personalized treatment, identification of non-invasive biomarkers for differentiation of tumor cellularity in PDAC would be clinically highly valuable. Here, we show that CA accumulation varies significantly in PDAC depending on tumor morphology, enabling non-invasive binary classification of tumor cellularity in vivo. In addition, our study reveals unappreciated effects of standard of care GEM treatment on CA accumulation that correlate with tumor vascularity and small molecule delivery. This finding has important implications for GEM-based combination treatment.

High tumor cellularity, non-invasively detected by diffusion weighted (DW)-MRI and CA accumulation in CT, has previously been identified as a predictor of poor outcome in PDAC [25, 26, 37, 41]. We further expand on this classification and suggest the biomarkers HUr, SI_t/a_, SI_t/m_, and AUC60r for a non-invasive stratification of tumor cellularity subgroups. Here, we show that both CT- and MRI-derived perfusion-related values can be used for patient stratification. In line with our observations, the particularly aggressive transcriptionally defined squamous PDAC subtype has been shown to have the highest difference between tumor and normal pancreas in CA accumulation and was associated with a higher number of secondary poor-prognosis mutations such as *TP53* or *SMAD4* and with consequently worse survival [26]. Moreover, the same study reported that stage IV patients revealed lower base line tumor CA accumulation compared to early stage operable patients [26]. In our study, the chemotherapy treated PDAC patient cohort also showed a lower base-line HUr value in comparison to the resected cohort, and murine tumors revealed decrease in AUC60r over time. These observations may be explained by the fact that as tumors progress, cellularity increases, which in turns leads to poorer vascularization and collapsed vessels due to high intra-tumoral pressure. Cumulative, these processes lead to reduced perfusion and CA accumulation in later stage PDAC, which is then reflected in lower HUr and AUC60r values. Therefore, routine CT- or MR-based non-invasive perioperative prediction of tumor cellularity by means of CA accumulation presents a widely applicable, biologically meaningful, and clinically relevant stratification strategy with great potential in routine patient care.

A direct correlation of in vivo imaging and ex vivo histopathology data is highly desirable yet challenging [40]. Especially in hPDAC, where more than 80% of patients are initially not eligible to surgery, imaging-correlated histopathology analyses are rare, and results are often obscured by the effects of neo-adjuvant chemotherapy. Here, we use GEMM of PDAC, which exhibit complex extracellular matrix on the background of an intact immune system, and recapitulate well the human condition. GEMM of PDAC were used in many meaningful preclinical trials [4, 5, 15, 16, 37, 38] with in-/ex-vivo sample co-registration [5, 37, 38, 40] and subsequent molecular tumor characterization [4, 5, 15, 16, 38, 42]. For example, manual co-registration of diverse imaging methods such as ultrasound, transmission electron microscopy, or DCE-MRI with corresponding histopathology allowed profound investigation of micro- and macro-vascular tumor architecture, treatment-induced vascular and stromal changes, and drug delivery in *KPC* tumors [5, 18, 42]. Moreover, endogenous mPDAC is, similar to hPDAC, highly resistant to GEM—only 5–10% of KPC tumors show response—allowing preclinical drug trials of high predictive value [18, 38, 42]. To fully leverage this potential, we used GEMM of different mutational backgrounds to optimally mirror the inter-tumoral heterogeneity that is noted in hPDAC [37] and found striking similarities in the behavior of the tumor perfusion-related biomarker AUC60r. Analogous to hPDAC, we observed substantial heterogeneity in CA enhancement in the analyzed mPDAC population: low cellularity tumors revealed high CA accumulation, whereas high cellularity tumors showed low CA accumulation. In addition, we provide a direct correlation of imaging-derived biomarker AUC60r with functionality of vascular compartment measured by the amount of open vessels calculated in the imaging-corresponding axial slice of the tumor tissue.

Reduced CA accumulation in PDAC, compared to the normal surrounding pancreas, has long been noted and presents one of the diagnostic clues in clinical routine patient management. Consequently, it has led to the introduction of therapies intended to normalize intra-tumoral pressure, vascularization, and drug delivery [43]. Indeed, therapy trials with stroma targeting agents have shown improved perfusion, monitored non-invasively by contrast enhanced ultrasound [4, 42] and DCE-MRI [18] in mPDAC as well as in hPDAC [30]. Subsequent reduced vascular collapse and increased drug delivery lead to prolonged survival of the treated animals [4, 5, 18, 42]. These studies confirm the high relevance of the vascular compartment and the potential role of perfusion-sensitive imaging derived biomarkers in treatment monitoring. Unfortunately, despite positive pre-clinical and early clinical trials, none of the stroma modifying agents has reached routine clinical application thus far [9, 11, 44].

Despite intermediate efficacy and a poor long-term outcome, GEM remains one of the standard of care treatment choices in hPDAC. Therefore, understanding its impact on tumor vasculature is an important interrogation. We observed a strong decrease in the DCE-MRI-derived perfusion-sensitive marker AUC60r following GEM-based treatment in mPDAC. This effect was most prominent in the initially well perfused mPDAC^low^ tumors. Human endothelial cells have been reported to be considerably more sensitive to GEM than are pancreatic tumor cells, both in vitro and in orthotopic xenotransplants [17]. In line with this observation, we noted a reduction in vascular proliferation in GEM-treated murine tumors. Other phenomena, such as apoptosis, were not analyzed, since GEM has been reported to specifically reduce proliferation in human and murine pancreatic tumor cells in vivo [42] and in human endothelial cells in vitro [17]. We further provide evidence that the reduction in vascular proliferation is of functional relevance, showing a reduced delivery of the systemically administered DNA-damaging substance cisplatin.

Our study also provides indirect evidence of the deteriorating effects of GEM on tumor vasculature in hPDAC by showing a significant decrease in CA accumulation in response to GEM-based, but not FOLFIRINOX-based, treatment. Similar to experiments in mPDAC, no significant differences between the treatments were observed in tumors with an initially poor CA accumulation. Nevertheless, in a subset of subjects, there was an increase in CA accumulation during GEM treatment, possibly related to low dosage (i.e., metronomic) delivery, which has previously been reported to normalize tumor vasculature by increased levels of the angiogenesis inhibitor thrombospondin-1 [45]. In addition, a direct effect of GEM on tumor cells reduces intra-tumoral pressure [19, 20] and likely increases tumor perfusion, which has previously been noted in GEM/Abraxane responders [29]. However, the latter study was also conducted in patients with advanced PDAC, and no histological correlation nor sub-grouping was performed.

There are several limitations to the interpretation of results of this work. The analyzed hPDAC cohort with available imaging-correlated tissue samples was small in size and our observations from it require further prospective testing. The differences in the baseline CA accumulation between human cohorts may reflect more advanced disease within the cohort of study h2, resulting in lower initial perfusion. Further biopsy- and imaging-guided neoadjuvant trials may provide the necessary information on that matter and help to adjust the proposed cutoff for patient stratification. CA accumulation is dependent on but not equivalent to perfusion, and therefore prospective trials including imaging-tissue correlation experiments are necessary to confirm or discard our hypothesis of GEM-induced perfusion effects in hPDAC. In addition, due to poor soft tissue contrast and limited time resolution of standard of care contrast CT in our mouse models, this work used different CA and acquisition methods in the human and murine imaging studies, which limits absolute comparability and direct translational impact. Nevertheless, the similar trends in patient-measured CT-derived HUr and MRI-derived SI_t/a_ values and murine perfusion related biomarkers support our hypothesis. Furthermore, a clinical study in patients with liver metastases of colorectal cancer confirmed that MRI biomarkers of vascular function, including iAUC60, correlate with structural features of tumor vessels detected by CT [22]. In addition, an improved technical setup for consecutive DCE-MRI and CT imaging of subcutaneous murine tumors was able to directly link functional MRI parameters to a structural CT-derived vascular volume parameter in vivo, which was confirmed by intra-vital microscopy and 3D ex vivo validation [22, 46].

In conclusion, we tested and applied two clinical imaging-derived perfusion-related biomarkers (AUC60r and HUr) for the non-invasive differentiation of cellularity related subgroups in murine and human PDAC. Applied to therapy response monitoring of PDAC, we observed a decrease in CA accumulation in response to GEM treatment. In addition, we provide evidence that GEM interferes with endothelial cell proliferation, further aggravating pre-existent hypo-perfusion of PDAC in vivo. Our observations thus suggest tumor CA accumulation as a biomarker for tumor stratification based on cellularity and for the longitudinal monitoring. Blockage of tumor neo-vascularization may partially explain the failure of gemcitabine-based combinatorial regiments previously observed in human PDAC trials.

## Supplementary Information

ESM 1 (PDF 3.26 mb)

## Data Availability

The datasets generated during and/or analyzed during the current study are available from the corresponding author on reasonable request.

## References

[1] Sung H, Ferlay J, Siegel RL, Laversanne M, Soerjomataram I, Jemal A, et al. Global cancer statistics 2020: GLOBOCAN estimates of incidence and mortality worldwide for 36 cancers in 185 countries. CA Cancer J Clin. 2021; 10.3322/caac.21660. 10.3322/caac.21660 33538338

[2] Society AC. Cancer Facts and Figures 2014-2021.

[3] Neoptolemos JP, Kleeff J, Michl P, Costello E, Greenhalf W, Palmer DH. Therapeutic developments in pancreatic cancer: current and future perspectives. Nat Rev Gastroenterol Hepatol. 2018;15:333–48. 10.1038/s41575-018-0005-x. 29717230 10.1038/s41575-018-0005-x

[4] Provenzano PP, Cuevas C, Chang AE, Goel VK, Von Hoff DD, Hingorani SR. Enzymatic targeting of the stroma ablates physical barriers to treatment of pancreatic ductal adenocarcinoma. Cancer Cell. 2012;21:418–29. 10.1016/j.ccr.2012.01.007. 22439937 10.1016/j.ccr.2012.01.007 PMC3371414

[5] Jacobetz MA, Chan DS, Neesse A, Bapiro TE, Cook N, Frese KK, et al. Hyaluronan impairs vascular function and drug delivery in a mouse model of pancreatic cancer. Gut. 2013;62:112–20. 10.1136/gutjnl-2012-302529. 22466618 10.1136/gutjnl-2012-302529 PMC3551211

[6] Hosein AN, Brekken RA, Maitra A. Pancreatic cancer stroma: an update on therapeutic targeting strategies. Nat Rev Gastroenterol Hepatol. 2020;17:487–505. 10.1038/s41575-020-0300-1. 32393771 10.1038/s41575-020-0300-1 PMC8284850

[7] Garcia-Sampedro A, Gaggia G, Ney A, Mahamed I, Acedo P. The state-of-the-art of phase II/III clinical trials for targeted pancreatic cancer therapies. J Clin Med. 2021;10 10.3390/jcm10040566. 10.3390/jcm10040566 PMC7913382 33546207

[8] Burris HA 3rd, Moore MJ, Andersen J, Green MR, Rothenberg ML, Modiano MR, et al. Improvements in survival and clinical benefit with gemcitabine as first-line therapy for patients with advanced pancreas cancer: a randomized trial. J Clin Oncol. 1997;15:2403–13. 10.1200/JCO.1997.15.6.2403. 9196156 10.1200/JCO.1997.15.6.2403

[9] Ramanathan RK, McDonough SL, Philip PA, Hingorani SR, Lacy J, Kortmansky JS, et al. Phase IB/II Randomized Study of FOLFIRINOX Plus Pegylated Recombinant Human Hyaluronidase Versus FOLFIRINOX Alone in Patients With Metastatic Pancreatic Adenocarcinoma: SWOG S1313. J Clin Oncol. 2019;37:1062–9. 10.1200/JCO.18.01295. 30817250 10.1200/JCO.18.01295 PMC6494359

[10] Catenacci DV, Junttila MR, Karrison T, Bahary N, Horiba MN, Nattam SR, et al. Randomized phase Ib/II study of gemcitabine plus placebo or vismodegib, a hedgehog pathway inhibitor, in patients with metastatic pancreatic cancer. J Clin Oncol. 2015;33:4284–92. 10.1200/JCO.2015.62.8719. 26527777 10.1200/JCO.2015.62.8719 PMC4678179

[11] Van Cutsem E, Tempero MA, Sigal D, Oh DY, Fazio N, Macarulla T, et al. Randomized phase III trial of pegvorhyaluronidase alfa with nab-paclitaxel plus gemcitabine for patients with hyaluronan-high metastatic pancreatic adenocarcinoma. J Clin Oncol. 2020;38:3185–94. 10.1200/JCO.20.00590. 32706635 10.1200/JCO.20.00590 PMC7499614

[12] Conroy T, Desseigne F, Ychou M, Bouche O, Guimbaud R, Becouarn Y, et al. FOLFIRINOX versus gemcitabine for metastatic pancreatic cancer. N Engl J Med. 2011;364:1817–25. 10.1056/NEJMoa1011923. 21561347 10.1056/NEJMoa1011923

[13] Von Hoff DD, Ervin T, Arena FP, Chiorean EG, Infante J, Moore M, et al. Increased survival in pancreatic cancer with nab-paclitaxel plus gemcitabine. N Engl J Med. 2013;369:1691–703. 10.1056/NEJMoa1304369. 24131140 10.1056/NEJMoa1304369 PMC4631139

[14] Koay EJ, Truty MJ, Cristini V, Thomas RM, Chen R, Chatterjee D, et al. Transport properties of pancreatic cancer describe gemcitabine delivery and response. J Clin Invest. 2014;124:1525–36. 10.1172/JCI73455. 24614108 10.1172/JCI73455 PMC3973100

[15] Buchholz SM, Goetze RG, Singh SK, Ammer-Herrmenau C, Richards FM, Jodrell DI, et al. Depletion of macrophages improves therapeutic response to gemcitabine in murine pancreas cancer. Cancers (Basel). 2020;12 10.3390/cancers12071978. 10.3390/cancers12071978 PMC7409345 32698524

[16] Hessmann E, Patzak MS, Klein L, Chen N, Kari V, Ramu I, et al. Fibroblast drug scavenging increases intratumoural gemcitabine accumulation in murine pancreas cancer. Gut. 2018;67:497–507. 10.1136/gutjnl-2016-311954. 28077438 10.1136/gutjnl-2016-311954 PMC5868285

[17] Laquente B, Lacasa C, Ginesta MM, Casanovas O, Figueras A, Galan M, et al. Antiangiogenic effect of gemcitabine following metronomic administration in a pancreas cancer model. Mol Cancer Ther. 2008;7:638–47. 10.1158/1535-7163.MCT-07-2122. 18347150 10.1158/1535-7163.MCT-07-2122

[18] Cao J, Pickup S, Clendenin C, Blouw B, Choi H, Kang D, et al. Dynamic contrast-enhanced MRI detects responses to stroma-directed therapy in mouse models of pancreatic Ductal Adenocarcinoma. Clin Cancer Res. 2019;25:2314–22. 10.1158/1078-0432.CCR-18-2276. 30587546 10.1158/1078-0432.CCR-18-2276 PMC6445712

[19] Cham KK, Baker JH, Takhar KS, Flexman JA, Wong MQ, Owen DA, et al. Metronomic gemcitabine suppresses tumour growth, improves perfusion, and reduces hypoxia in human pancreatic ductal adenocarcinoma. Br J Cancer. 2010;103:52–60. 10.1038/sj.bjc.6605727. 20531411 10.1038/sj.bjc.6605727 PMC2905290

[20] Yapp DT, Wong MQ, Kyle AH, Valdez SM, Tso J, Yung A, et al. The differential effects of metronomic gemcitabine and antiangiogenic treatment in patient-derived xenografts of pancreatic cancer: treatment effects on metabolism, vascular function, cell proliferation, and tumor growth. Angiogenesis. 2016;19:229–44. 10.1007/s10456-016-9503-z. 26961182 10.1007/s10456-016-9503-z PMC4819514

[21] Hamdy A, Ichikawa Y, Toyomasu Y, Nagata M, Nagasawa N, Nomoto Y, et al. Perfusion CT to assess response to neoadjuvant chemotherapy and radiation therapy in pancreatic ductal adenocarcinoma: initial experience. Radiology. 2019;292:628–35. 10.1148/radiol.2019182561. 31287389 10.1148/radiol.2019182561

[22] Kannan P, Kretzschmar WW, Winter H, Warren D, Bates R, Allen PD, et al. Functional parameters derived from magnetic resonance imaging reflect vascular morphology in preclinical tumors and in human liver metastases. Clin Cancer Res. 2018;24:4694–704. 10.1158/1078-0432.CCR-18-0033. 29959141 10.1158/1078-0432.CCR-18-0033 PMC6171743

[23] Rajendran R, Huang W, Tang AM, Liang JM, Choo S, Reese T, et al. Early detection of antiangiogenic treatment responses in a mouse xenograft tumor model using quantitative perfusion MRI. Cancer Med. 2014;3:47–60. 10.1002/cam4.177. 24403176 10.1002/cam4.177 PMC3930389

[24] Nebuloni L, Kuhn GA, Vogel J, Muller R. A novel in vivo vascular imaging approach for hierarchical quantification of vasculature using contrast enhanced micro-computed tomography. PLoS One. 2014;9:e86562. 10.1371/journal.pone.0086562. 24475146 10.1371/journal.pone.0086562 PMC3903581

[25] Torphy RJ, Wang Z, True-Yasaki A, Volmar KE, Rashid N, Yeh B, et al. Stromal content is correlated with tissue site, contrast retention, and survival in pancreatic adenocarcinoma. JCO Precis Oncol. 2018;2018 10.1200/PO.17.00121. 10.1200/PO.17.00121 PMC6262879 30506016

[26] Koay EJ, Lee Y, Cristini V, Lowengrub JS, Kang Y, Lucas FAS, et al. A visually apparent and quantifiable CT imaging feature identifies biophysical subtypes of pancreatic ductal adenocarcinoma. Clin Cancer Res. 2018;24:5883–94. 10.1158/1078-0432.CCR-17-3668. 30082477 10.1158/1078-0432.CCR-17-3668 PMC6279613

[27] Jungmann F, Kaissis GA, Ziegelmayer S, Harder F, Schilling C, Yen HY, et al. Prediction of tumor cellularity in resectable PDAC from preoperative computed tomography imaging. Cancers (Basel). 2021;13 10.3390/cancers13092069. 10.3390/cancers13092069 PMC8123300 33922981

[28] Tofts PS, Brix G, Buckley DL, Evelhoch JL, Henderson E, Knopp MV, et al. Estimating kinetic parameters from dynamic contrast-enhanced T(1)-weighted MRI of a diffusable tracer: standardized quantities and symbols. J Magn Reson Imaging. 1999;10:223–32. 10.1002/(SICI)1522-2586(199909)10:33.0.CO;2-S. 10508281 10.1002/(sici)1522-2586(199909)10:3<223::aid-jmri2>3.0.co;2-s

[29] Tang W, Liu W, Li HM, Wang QF, Fu CX, Wang XH, et al. Quantitative dynamic contrast-enhanced MR imaging for the preliminary prediction of the response to gemcitabine-based chemotherapy in advanced pancreatic ductal carcinoma. Eur J Radiol. 2019;121:108734. 10.1016/j.ejrad.2019.108734. 31743881 10.1016/j.ejrad.2019.108734

[30] Pijnappel EN, Wassenaar NPM, Gurney-Champion OJ, Klaassen R, van der Lee K, Pleunis-van Empel MCH, et al. Phase I/II study of LDE225 in combination with gemcitabine and nab-paclitaxel in patients with metastatic pancreatic cancer. Cancers (Basel). 2021;13 10.3390/cancers13194869. 10.3390/cancers13194869 PMC8507646 34638351

[31] Kinh Do R, Reyngold M, Paudyal R, Oh JH, Konar AS, LoCastro E, et al. Diffusion-weighted and dynamic contrast-enhanced MRI derived imaging metrics for stereotactic body radiotherapy of pancreatic ductal adenocarcinoma: preliminary findings. Tomography. 2020;6:261–71. 10.18383/j.tom.2020.00015. 32548304 10.18383/j.tom.2020.00015 PMC7289241

[32] Klaassen R, Steins A, Gurney-Champion OJ, Bijlsma MF, van Tienhoven G, Engelbrecht MRW, et al. Pathological validation and prognostic potential of quantitative MRI in the characterization of pancreas cancer: preliminary experience. Mol Oncol. 2020;14:2176–89. 10.1002/1878-0261.12688. 32285559 10.1002/1878-0261.12688 PMC7463316

[33] Khalifa F, Soliman A, El-Baz A, Abou El-Ghar M, El-Diasty T, Gimel'farb G, et al. Models and methods for analyzing DCE-MRI: a review. Med Phys. 2014;41:124301. 10.1118/1.4898202. 25471985 10.1118/1.4898202

[34] Chen CFHL, Lui CC, Lee CC, Weng HH, Tsai YH, Liu HL. In vivo correlation between semi-quantitative hemodynamic parameters and ktrans derived from DCE-MRI of brain tumors. Inc Int J Imaging Syst Technol. 2012;22:132–6. 10.1002/ima.22013.

[35] Steingoetter A, Svensson J, Kosanke Y, Botnar RM, Schwaiger M, Rummeny E, et al. Reference region-based pharmacokinetic modeling in quantitative dynamic contract-enhanced MRI allows robust treatment monitoring in a rat liver tumor model despite cardiovascular changes. Magn Reson Med. 2011;65:229–38. 10.1002/mrm.22589. 20872863 10.1002/mrm.22589

[36] Mendler CT, Feuchtinger A, Heid I, Aichler M, D'Alessandria C, Pirsig S, et al. Tumor uptake of anti-CD20 Fabs depends on tumor perfusion. J Nucl Med. 2016;57:1971–7. 10.2967/jnumed.116.176784. 27417649 10.2967/jnumed.116.176784

[37] Heid I, Steiger K, Trajkovic-Arsic M, Settles M, Esswein MR, Erkan M, et al. Co-clinical assessment of tumor cellularity in pancreatic cancer. Clin Cancer Res. 2017;23:1461–70. 10.1158/1078-0432.Ccr-15-2432. 27663591 10.1158/1078-0432.CCR-15-2432

[38] Trajkovic-Arsic M, Heid I, Steiger K, Gupta A, Fingerle A, Worner C, et al. Apparent Diffusion Coefficient (ADC) predicts therapy response in pancreatic ductal adenocarcinoma. Sci Rep. 2017;7:17038. 10.1038/s41598-017-16826-z. 29213099 10.1038/s41598-017-16826-z PMC5719052

[39] Braren R, Curcic J, Remmele S, Altomonte J, Ebert O, Rummeny EJ, et al. Free-breathing quantitative dynamic contrast-enhanced magnetic resonance imaging in a rat liver tumor model using dynamic radial T(1) mapping. Invest Radiol. 2011;46:624–31. 10.1097/RLI.0b013e31821e30e7. 21577121 10.1097/RLI.0b013e31821e30e7

[40] Ballke S, Heid I, Mogler C, Braren R, Schwaiger M, Weichert W, et al. Correlation of in vivo imaging to morphomolecular pathology in translational research: challenge accepted. EJNMMI Res. 2021;11:83. 10.1186/s13550-021-00826-2. 34453623 10.1186/s13550-021-00826-2 PMC8401369

[41] Winter JM, Ting AH, Vilardell F, Gallmeier E, Baylin SB, Hruban RH, et al. Absence of E-cadherin expression distinguishes noncohesive from cohesive pancreatic cancer. Clin Cancer Res. 2008;14:412–8. 10.1158/1078-0432.CCR-07-0487. 18223216 10.1158/1078-0432.CCR-07-0487 PMC3810144

[42] Olive KP, Jacobetz MA, Davidson CJ, Gopinathan A, McIntyre D, Honess D, et al. Inhibition of Hedgehog signaling enhances delivery of chemotherapy in a mouse model of pancreatic cancer. Science. 2009;324:1457–61. 10.1126/science.1171362. 19460966 10.1126/science.1171362 PMC2998180

[43] Matuszewska K, Pereira M, Petrik D, Lawler J, Petrik J. Normalizing tumor vasculature to reduce hypoxia, enhance perfusion, and optimize therapy uptake. Cancers (Basel). 2021;13 10.3390/cancers13174444. 10.3390/cancers13174444 PMC8431369 34503254

[44] Ko AH, LoConte N, Tempero MA, Walker EJ, Kate Kelley R, Lewis S, et al. A phase I study of FOLFIRINOX plus IPI-926, a hedgehog pathway inhibitor, for advanced pancreatic adenocarcinoma. Pancreas. 2016;45:370–5. 10.1097/MPA.0000000000000458. 26390428 10.1097/MPA.0000000000000458 PMC5908466

[45] Mpekris F, Baish JW, Stylianopoulos T, Jain RK. Role of vascular normalization in benefit from metronomic chemotherapy. Proc Natl Acad Sci USA. 2017;114:1994–9. 10.1073/pnas.1700340114. 28174262 10.1073/pnas.1700340114 PMC5338413

[46] Kersemans V, Kannan P, Beech JS, Bates R, Irving B, Gilchrist S, et al. Improving in vivo high-resolution CT imaging of the tumour vasculature in xenograft mouse models through reduction of motion and bone-streak artefacts. PLoS ONE. 2015;10:e0128537. 10.1371/journal.pone.0128537. 26046526 10.1371/journal.pone.0128537 PMC4457787

