# Correction to: Functional biomarkers derived from computed tomography and magnetic resonance imaging differentiate PDAC subgroups and reveal gemcitabine‑induced hypo‑vascularization

**DOI:** 10.1007/s00259-025-07195-1

**Published:** 2025-03-18

**Authors:** Irina Heid, Marija Trajkovic-Arsic, Fabian Lohöfer, Georgios Kaissis, Felix N. Harder, Moritz Mayer, Geoffrey J. Topping, Friderike Jungmann, Barbara Crone, Moritz Wildgruber, Uwe Karst, Lucia Liotta, Hana Algül, Hsi-Yu Yen, Katja Steiger, Wilko Weichert, Jens T. Siveke, Marcus R. Makowski, Rickmer F. Braren

**Affiliations:** 1 School of Medicine, Institute of Diagnostic and Interventional Radiology, Technical University of Munich, Munich, Germany; 2 Division of Solid Tumor Translational Oncology, German Cancer Consortium (DKTK, partner site Essen) and German Cancer Research Center, DKFZ, Heidelberg, Germany; 3 Bridge Institute of Experimental Tumor Therapy, West German Cancer Center, University Hospital Essen, Essen, Germany; 4 Department of Computing, Imperial College London, London, SW7 2AZ UK; 5 School of Medicine, Institute for Artificial Intelligence in Medicine and Healthcare, Technical University of Munich, Munich, Germany; 6 School of Medicine, Department of Nuclear Medicine, Technical University of Munich, Munich, Germany; 7 Institute of Inorganic and Analytical Chemistry, University of Muenster, Muenster, Germany; 8 Institute of Clinical Radiology, University Hospital Muenster, Muenster, Germany; 9 Department of Radiology, University Hospital, LMU Munich, Munich, Germany; 10 School of Medicine, Clinic and Policlinic of Internal Medicine II, Technical University of Munich, Munich, Germany; 11 Comprehensive Cancer Center München, Chair for Tumor Metabolism, Klinikum rechts der Isar, Technical University of Munich, School of Medicine and Health, Munich, Bavaria Germany; 12 School of Medicine, Institute of Pathology, Technical University of Munich, Munich, Germany; 13 German Cancer Consortium (DKTK, partner Site Munich), Munich, Germany

**Correction to: European Journal of Nuclear Medicine and Molecular Imaging (2022) 50:115–129**

10.1007/s00259-022-05930-6

The authors regret that the affiliation for Dr. Hana Algül in the original published article is incorrect.

The incorrect affiliation is: Comprehensive Cancer Center Munich at the Klinikum rechts der Isar (CCCMTUM), Technical University of Munich, Munich, Germany

The correct affiliation is: Comprehensive Cancer Center München, Chair for Tumor Metabolism, Klinikum rechts der Isar, Technical University of Munich, School of Medicine and Health, Munich, Bavaria, Germany

The original article has been corrected.

