# Supplementary material for: Functional biomarkers derived from computed tomography and magnetic resonance imaging differentiate PDAC subgroups and reveal gemcitabine-induced hypo-vascularization
Source: Eur J Nucl Med Mol Imaging. 2022 Sep 8;50(1):115–29. doi: 10.1007/s00259-022-05930-6 (PMC9668793; doi:10.1007/s00259-022-05930-6)
Supplement: Supplementary file 1 — (PDF 3.26 mb) [file 259_2022_5930_MOESM1_ESM.pdf]

Functional biomarkers derived from computed tomography and magnetic resonance imaging differentiate PDAC subgroups and reveal gemcitabine-induced hypo-vascularization

Irina Heid[1], Marija Trajkovic-Arsic[2,3], Fabian Lohöfer[1], Georgios Kaissis[1,4, 5], Felix N. Harder[1], Moritz Mayer[1], Geoffrey J. Topping[6], Friederike Jungmann[1], Barbara Crone[7], Moritz Wildgruber[8,9], Uwe Karst[7], Lucia Liotta[10], Hana Algül[11], Hsi-Yu Yen[12], Katja Steiger[12], Wilko Weichert[12, 13], Jens T. Siveke[2,3], Marcus R. Makowski[1] and Rickmer F. Braren[1, 13]

Corresponding authors

Dr. rer. nat. Irina Heid

E-mail: Irina.Heid@tum.de

Prof. Dr. Rickmer Braren, MD

E-mail: rbraren@tum.de

Technical University of Munich, School of Medicine

Institute of Diagnostic and Interventional Radiology

Ismaninger Str. 22, D-81675 Munich, Germany

Supplementary methods:

CT and MRI analysis in patients

For the analysis of patients CT datasets, mean tumor enhancement in the portal venous (PV) phase was calculated from a 5 mm region of interest (ROI) placed in the tumor in axial 3 mm images or image reconstructions, avoiding areas of necrosis or ambiguity due to partial volume effects. The same procedure was carried out for the aorta, avoiding areas of calcifications, infiltration or partial volume effects. ROI placement was undertaken by consensus of two abdominal radiologists (FL, GK) with training in pancreatic image analysis and experience of 4 and 6 years. Readers were allowed to window all images as needed to guarantee correct positioning of the ROI. Consensus was reached in all cases. Relative enhancement in the tumor compared to aorta (HUr) in the PV phase was recorded for all scans and used for further analysis. The analysis of patients MRI datasets was performed in the same manner as described above in the CT analysis, while additionally pacing an ROI into the muscle for comparison with murine studies. The analysis was performed on T_1-weighted images with spectral fat saturation acquired in the early venous phase between 50-70 seconds and late venous phase between 100-180 seconds after administration of CA.

For the assessment of tumor response under chemotherapy, longest diameter measurements of the tumors were taken, as stipulated under the Response Evaluation Criteria in Solid Tumors (RECIST) guidelines and the change in longest diameter under chemotherapy was recorded.

DCE-MR-Imaging protocol and imaging data analysis in mice

Before imaging, mice were anaesthetized by continuous gaseous infusion of 1.5% - 3% isoflurane (Abbott GmbH, Wiesbaden, Germany) using a veterinary anesthesia System (Vetland Medical Sales and Services, Louisville, KY, USA). During all imaging experiments, animal temperature was maintained and continuously monitored and eyes were protected with an eye ointment. After fixation of a tail vein catheter for bolus injections, animals were placed onto a 47 mm microscopy surface coil inside the clinical 1.5 T MRI System (Achieva 1.5 T, Philips Medical Systems, Best, The Netherlands). An axial multi-slice T_2-weighted turbo spin echo (TSE) sequence (resolution 0.3 x 0.3 x 0.7 mm^3, minimum 35 slices, TE = 90 ms, TR > 3 s) was acquired for anatomical reference, tumor detection and tumor volume quantification. The subsequent DCE-MRI experiments and data analyses were performed during free breathing using a previously described fast single-shot Look-Locker based radial T_1 mapping technique using the golden cut principle (LLGC) [35, 36, 39]. In brief, a bolus dose of 0.04 mmol/kg of Gd-DTPA (Magnevist®) was administered after 60 s. Dynamic T_1 mapping of one axial slice positioned over the tumor(s) and spinal muscles was performed every 6 s for a period of 10 min (resolution = 0.71 x 0.71 x 2 mm^3, TR/TE = 12.6/5.7 ms, radial profiles = 206, flip angle = 10°, Tacq = 2.6 s, Tpause = 3.4 s).

After imaging, ROIs were manually defined to select tumor or muscle areas and mean ROI T_1 values were calculated over time. Histologically confirmed areas of necrosis were excluded from all functional imaging analyses. MRI data analysis was performed using either Osirix Imaging Software or in-house software written in IDL (ITT, Boulder, CO). For DCE-MRI analyses, the areas under the concentration curve until 60 s of vital tumor and spinal muscle (AUC60r) were computed using the trapezoidal rule. The tumor to muscle ratios were then calculated for statistical comparisons and presented as AUC60r.

Tissue preparation, histological staining and analysis

After removal, whole mouse abdomen containing organs of interest were fixed in 4% PFA/PBS solution, dehydrated and paraffin embedded (FFPE) in one block according to standard pathology core conditions of Technical University of Munich and the protocol described in [40]. At least 10 FFPE slices of 2-4 µm per animal, cut every 200-400 µm, were stained (Mayer's Haemalaun and Eosin (H&E), according to a standard protocol) for tissue visualization and manual correlation with in vivo imaging. Histopathological grading, regional amount of stroma and of cancer cells were evaluated manually by two experienced pathologists (KS, H-YY). For visualization of murine vessel architecture, 1-3 imaging-correlated FFPE slices were immunohistochemically (IHC) stained with antibodies against CD31: Abcam Cat# ab28364, RRID:AB_726362 (1:100; study m1, m2), Dianova Cat# DIA-310, RRID:AB_2631039 (1:100; study m3, m4) and anti CD34 (1:200, clone QBEnd/10, BSB5230) using a BenchMark XT automated stainer (Ventana, Tucson, AZ) or Bond RXm system (Leica Biosystems, Wetzlar, Germany). Antibody binding was detected using DAB as chromogen after pre-incubation with respectively EnVision rabbit (Agilent), HRP Universal Multimer (Ventana) or a polymer refine detection kit without post primary reagent (Leica). Nuclei were counterstained with haemalaun. Vascular proliferation was determined by double staining of CD31 (Dianova, described above) and Ki67 (1:200, NeoMarkers/LabVision Corporation, RM-9106-S0) according to sequential staining procedure on Bond RXm system, where ki67 was visualized with Fast Red as chromogen. All micrographs were made using an Aperio Scanner (Leica). Open and total vessel amount as well as proliferation were calculated manually in 5-10 20x magnification images per tumor region, depending on the tumor region size.

Laser Ablation – Inductively Coupled Plasma – Mass Spectrometry (LA-ICP-MS) imaging

Upon cisplatin injection as detailed above, tissues were perfused with 0.9% NaCl and 4% paraformaldehyde in phosphate-based solution (PFA/PBS). Pancreatic tumors and other organs were sliced in up to 3 slices and either snap frozen in liquid nitrogen or fixed in 4% PFA/PBS solution. Snap frozen mouse pancreas samples were sectioned in 10 µm thin sections using a cryomicrotome (CryoStar NX70, Thermo) operated at -23 °C and thaw-mounted on microscopic glass slides. LA-ICP-MS experiments were carried out using a laser ablation system LSX-213 (CETAC) with a wavelength of

213 nm and Chromium 2.2 software. The laser system device was coupled to a triple quadrupole based inductively coupled plasma mass spectrometer (iCap TQ, Thermo Fisher Scientific, Germany). The LA parameters regarding spot size, laser energy, scanning speed and carrier gas flow were optimized based on the best signal-to-noise ratio in combination with highest spatial resolution. The selected samples were ablated using a line by line scan (0 µm gap) with a spot size of 50 µm, a scan rate of 100 µm/s, a laser energy density of 7.55 J/cm^2 and laser repetition frequency of 20 Hz. The laser generated aerosol was transported by a carrier gas of helium (0.8 L/min) to the ICP-MS. For maximum sensitivity and to minimize possible interferences, the measurement was performed in kinetic energy discrimination mode (KED) with helium as cell gas. The isotope ^{195}Pt was monitored. Data evaluation for distribution maps was performed using the software ImageJ (National Institutes of Health, USA).

For platinum quantification by LA-ICP-MS, matrix-matched standards based on gelatin were prepared, with defined platinum concentrations (0, 0.05, 0.1, 0.5, 1.0, 5.0 and 10.0 mg/kg). To convert measured intensities in concentrations, the calibration function (linear regression of averaged ion intensities for the respective standard concentration) was determined and applied using Microsoft Excel 2010 (Microsoft, Redmond, USA). Further image processing was carried out with ImageJ (National Institute of Health, Northampton, USA). For image generation, the concentration matrices were transferred with a color code to distribution images. To determine the mean platinum concentration in the investigated tumor regions, the areas of interest were defined in the micrograph and evaluated in the respective platinum image.

Supplementary figures:

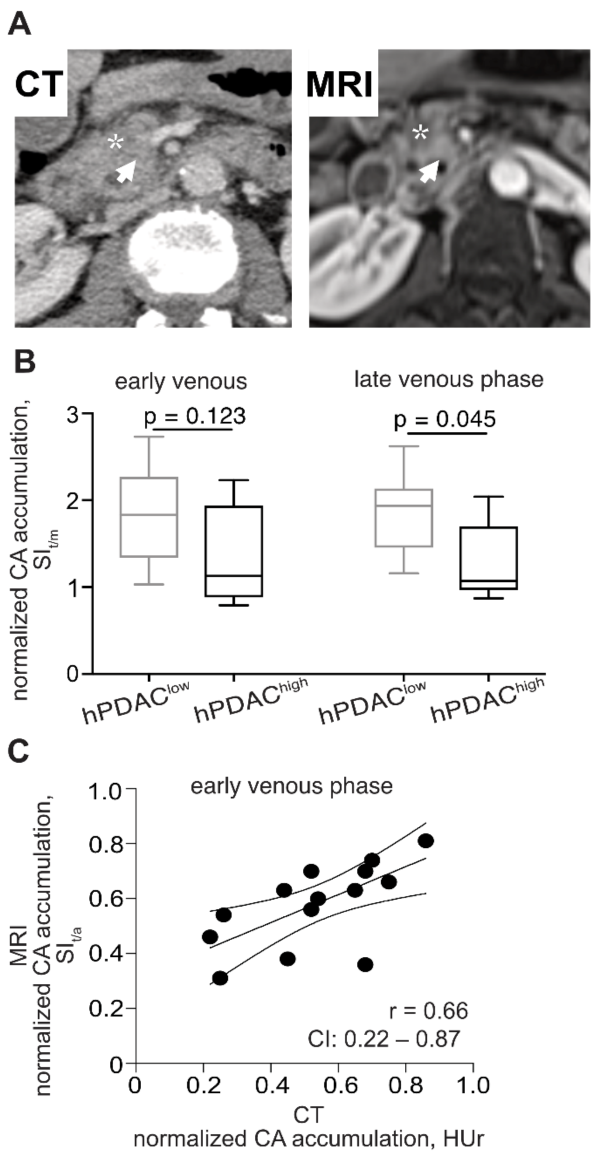

Supplementary Figure 1: **Correlation of CT and MRI CA accumulation in human (h)PDAC.** (**A**) Intra-tumoral differences in CA enhancement pattern of $hPDAC^{low}$ (arrow) and $hPDAC^{high}$ (star) detected in preoperative axial CT (Ultravist®-370) and DCE-MRI (Magnevist®, T_1-weighted with spectral fat-saturation). All images were acquired 70 s after administration of contrast agent. (**B**) Box-and-whisker plot of CA accumulation values for histologically confirmed $hPDAC^{low}$ and $hPDAC^{high}$ regions derived from early (70 s) and late (100-180 s) venous phase of DCE-MRI calculated as signal intensity of tumor to muscle ratios ($SI_{t/m}$). (**C**) Correlation of normalized CA accumulation at 70 s post injection estimated as signal intensity of tumor to aorta ratios ($SI_{t/a}$) on DCE-MRI and HUr on CT (p = 0.008).

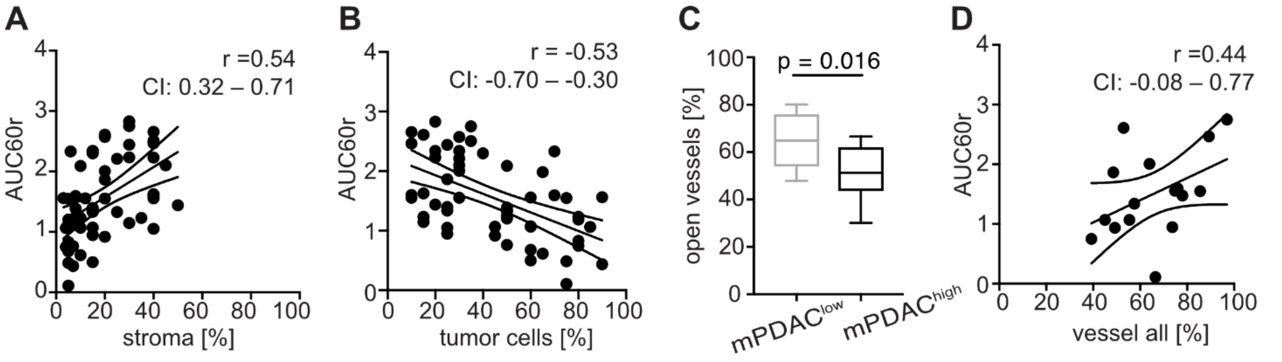

<u>Supplementary Figure 2</u>: Correlations of tumor CA accumulation parameter AUC60r with per cent of stroma (A) and per cent of tumor cells (B), tumor cellularity groups (C) and per cent of overall amount of vessels (D) within the region of interest in mPDAC.

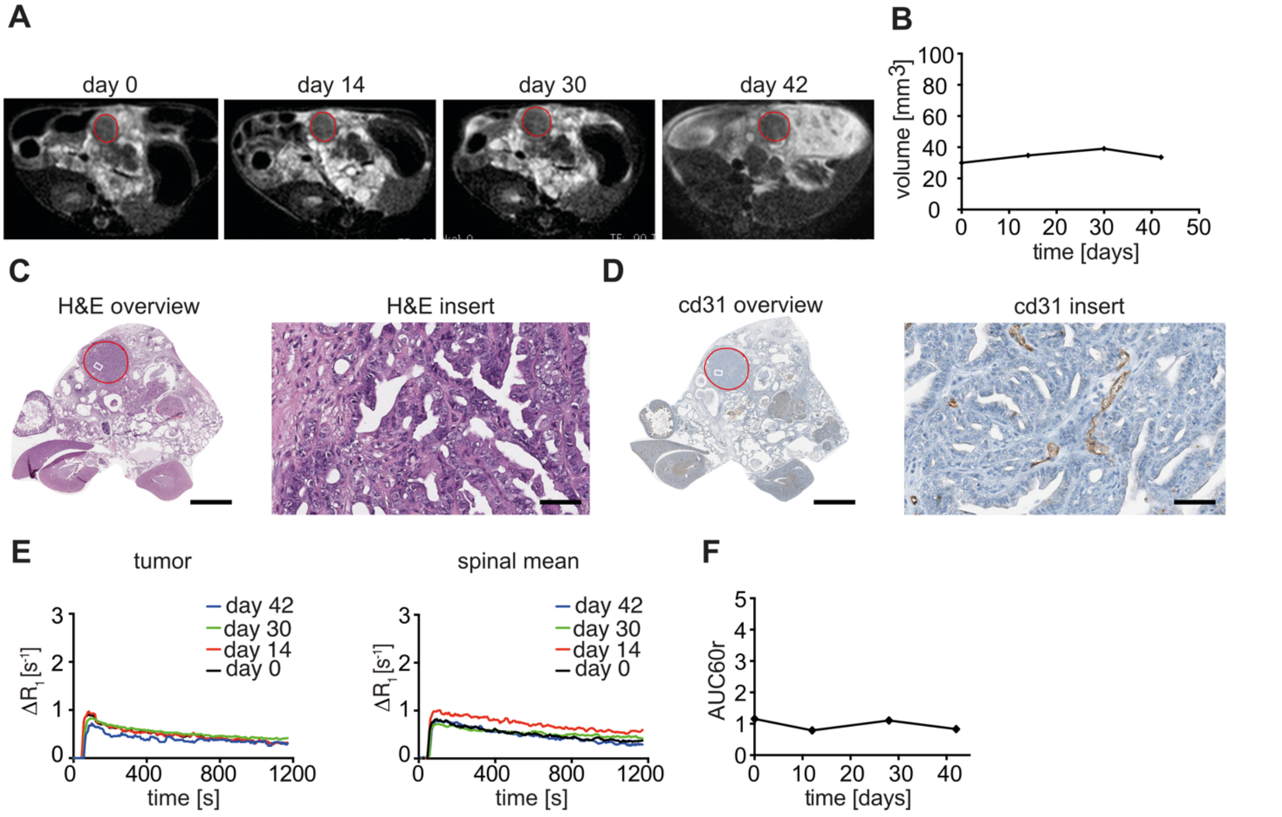

<u>Supplementary Figure 3</u>: Representative example of a stable mPDAC tumor showing no change in CA accumulation over time. (**A**) T_2-weighted images showing the same tumor (circular roi) over a period of 48 days. (**B**) Volume analysis of the solid part of the tumor. (**C**) Corresponding photomicrographs of H&E stained overview (scale bar 1 mm) and regional magnification (scale bar 50 µm). (**D**) Corresponding photomicrographs of CD31 stained overview (scale bar 1 mm) and regional magnification (scale bar 50 µm). (**E**) ΔR_1 time curves of tumor (left panel) and mean spinal muscle (middle panel) as well as AUC60r of the tumor over time (**F**).

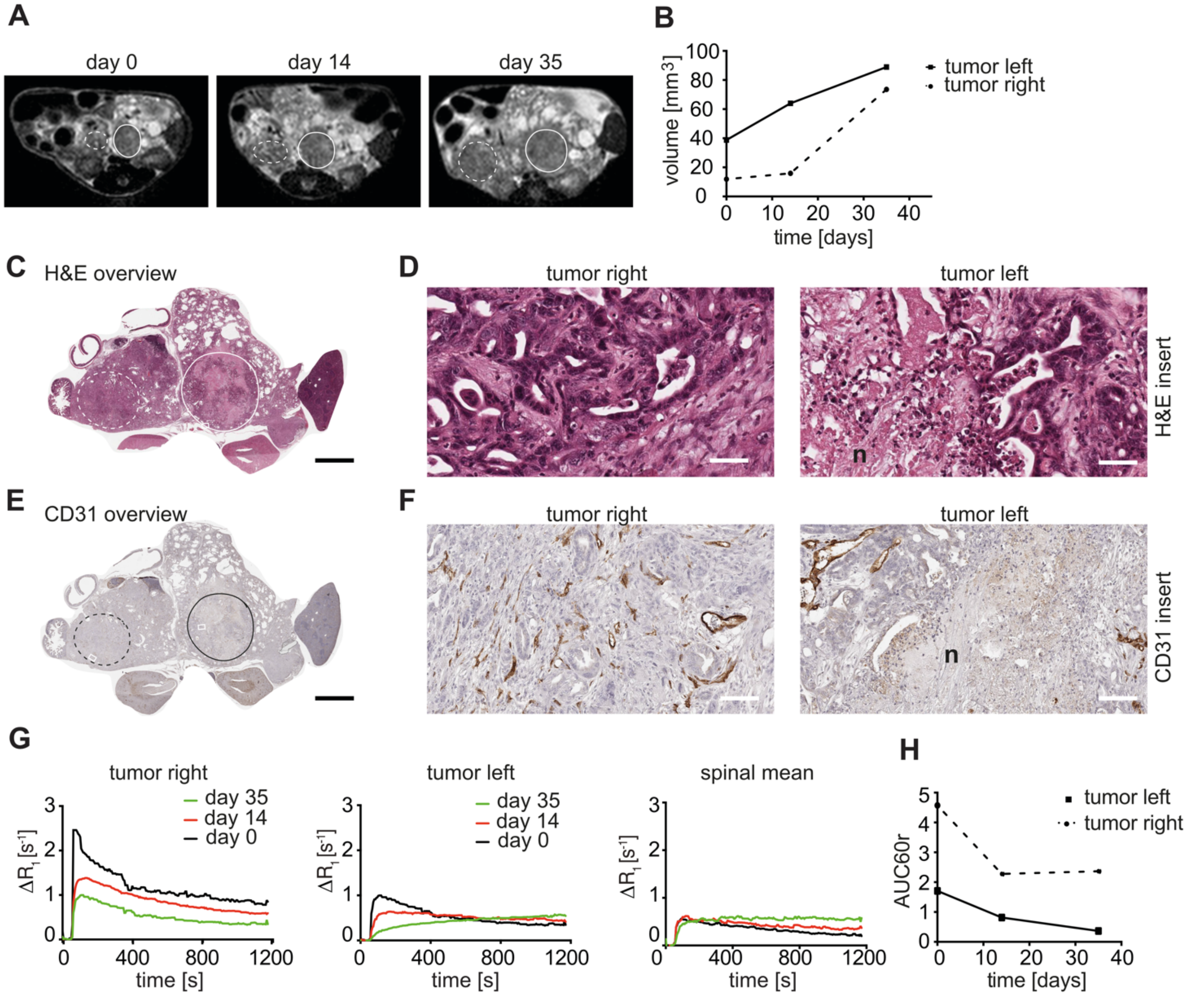

<u>Supplementary Figure 4:</u> Representative example of two mPDAC tumors showing changes in CA accumulation over time. (**A**) T_2-weighted images showing tumor left (solid line) and tumor right (dotted line) over a period of 35 days. (**B**) Volume analysis of the solid part of both tumors. (**C**) Corresponding photomicrographs of H&E stained overview (scale bar 1mm) and (**D**) regional magnification (scale bar 50 µm, n = necrosis). (**E**) Corresponding photomicrographs of CD31 stained overview (scale bar 1 mm) and (**F**) regional magnification (scale bar 50 µm). (**G**) ΔR_1 time curves of tumor right (left panel), tumor left (middle panel) and mean spinal muscle (right panel) as well as AUC60r tumor to muscle ratio of the tumors over time (**H**). Note that tumor left shows no peak on the day 35, reflecting abundant necrosis.

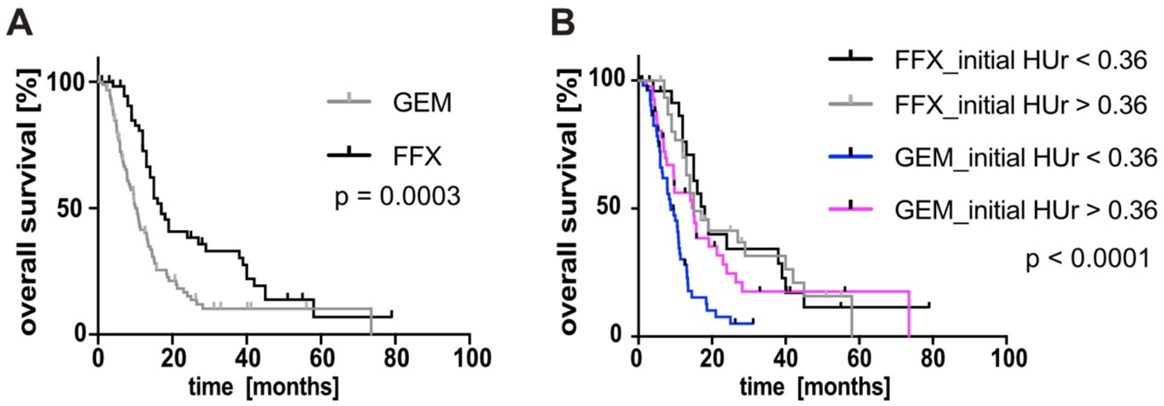

<u>Supplementary Figure 5:</u> **Overall survival analysis of hPDAC in chemotherapy treated patient cohort.**
